# Adapting Wine Grape Ripening to Global Change Requires a Multi-Trait Approach

**DOI:** 10.3389/fpls.2021.624867

**Published:** 2021-02-05

**Authors:** Bruno Suter, Agnes Destrac Irvine, Mark Gowdy, Zhanwu Dai, Cornelis van Leeuwen

**Affiliations:** ^1^EGFV, Univ. Bordeaux, Bordeaux Sciences Agro, INRAE, ISVV, F-33882, Villenave d’Ornon, France; ^2^Beijing Key Laboratory of Grape Science and Enology, CAS Key Laboratory of Plant Resources, Institute of Botany, Chinese Academy of Sciences, Beijing, China

**Keywords:** grapevine cultivars, berry sugar accumulation traits, phenotypic plasticity, climate change, genotype-environment interaction, modeling

## Abstract

In winegrowing regions around the world increasing temperature associated with climate change is responsible for earlier harvests and is implicated in undesirably high sugar concentrations at harvest. Determining the suitability of grapevine varieties in existing or new winegrowing areas has often been based on temperature, without considering other factors. The purpose of this study was to quantify key berry sugar accumulation traits and characterize their plasticity in response to several climate variables. Data was collected from 36 different cultivars over 7 years (2012–2018) from an experimental vineyard in Bordeaux, France. Sugar amounts were obtained through weekly berry sampling starting at mid-veraison and continuing until after technological maturity. The variation in sugar accumulation traits for all cultivars, when considered together, were well explained by cultivar, year, and their interaction, highlighting the relative roles of genetic variation and phenotypic plasticity. Sugar accumulation traits were affected by antecedent and concurrent climate factors such as photosynthetically active radiation, temperature, and vine water status, whether before, or after mid-veraison. In addition, other traits such as berry weight at mid-veraison and date of mid-veraison had an important influence on sugar accumulation traits. More notably, the relative importance of these factors varied significantly by cultivar. The specific physiological mechanisms driving the plasticity of these traits remain to be identified. Adaptation to climate change cannot be based on temperature alone and crop responses cannot be generalized across genotypes, even within species.

## Introduction

Wine grape (*Vitis vinifera* L.) vineyards covered more than 7.4 million hectares worldwide (OIV, 2018). As of 2015, the estimated net worth of the wine industry was more than 258 billion euros (Lecat et al., 2019). Winegrowers have classically selected different cultivars of wine grapes for the phenotypic traits that best match their (micro-)climates (Wolkovich et al., 2018) and soils. They maintain those cultivars that produce consistent yields and reach appropriate balance of sugar, acid, and other compounds under local climatic conditions (van Leeuwen and Seguin, 2006).

A major concern is that crop yields and quality may be significantly affected by climate change (Fraga et al., 2012). It is expected that temperatures will increase and drought will intensify in many regions across the globe (Intergovernmental Panel on Climate Change [IPCC], 2014). Climatic conditions during grape ripening have already been affected, resulting in altered grape composition at harvest (Fraga et al., 2012). Grapes are being harvested at increasingly higher sugar levels, resulting in wines with increased alcohol levels (Duchène and Schneider, 2005; Godden et al., 2015).

Sugar is one of the most important metabolites in grape berries used for wine production. Not only is the sugar concentration a major driver of the alcohol level of the finished wine, its levels during berry ripening are also involved in regulating development of the phenolic compounds that give color, flavor, and tannin structure to the wine (Conde et al., 2007). Duration of sugar loading may also be of interest to growers concerned with achieving adequate phenolic maturity of the grapes (Deloire et al., 2005).

Moving viticultural production to areas with more suitable climates may lead to conflicts in land use and freshwater ecosystems (Hannah et al., 2013). In addition, the land previously cultivated with grapevines may be unsuitable for other types of agriculture. A more sustainable adaptation, which can readily be implemented by winegrowers, would be to change to cultivars that are better suited to their objectives as the climate warms. To assess their adaptability, however, it is necessary to identify key ripening traits and their plasticity under different environmental conditions for a wide range of those cultivars.

Sugar accumulation during ripening is the net sum of sugar loading into, and sugar metabolism within the berry, with changes in the berry water balance affecting concentration. This ripening process starts at veraison, concurrent with berry softening and color change and can be subsequently affected by environmental conditions and vineyard management practices (Dai et al., 2009). The timing of veraison is a trait that can indirectly affect sugar accumulation. It drives the start of ripening and may be a factor in determining both the length of time available for ripening and the climatic conditions that the vine and berries will experience during ripening. The veraison date is influenced by both cultivar genetics and environmental conditions prior to veraison, and can be considered as a proxy for those conditions (Parker et al., 2011). For growers, both the earliness of the cultivar and its sugar accumulation traits are important considerations in assessing whether a given cultivar is adapted to their local climatic conditions.

The trajectory of sugar accumulation in grape berries follows a sigmoidal pattern with slow accumulation at the onset of veraison, rapid accumulation just after veraison and several weeks later reaching a plateau phase (Coombe and McCarthy, 2000). At the end of the ripening period, sugar content per berry no longer increases, but sugar concentration may continue to increase due to berry dehydration (Keller et al., 2016) or decrease due to dilution. Several researchers have already captured the dynamics of sugar accumulation through modeling approaches that estimate the rate of sugar accumulation, the amount of sugar at maturity and the timing of the plateau phase (Parker et al., 2020). This information is available, however, for only a few grape cultivars. Also, direct comparison of results from different studies may be difficult due to differences in experimental conditions, such as soils and climate.

Sugar accumulation traits have been found to be influenced by climatic variables, such as average temperature, photosynthetically active radiation (PAR), and water availability, with the effect depending on whether it was experienced during berry development pre-veraison, or post-veraison (Jones and Davis, 2000). Temperature was found to have an important effect on the rate and the total content of sugar accumulated (Greer and Weedon, 2014), but a relatively small effect on final sugar concentrations (Coombe, 1987) and corresponding rates of concentration increase (Sadras and Petrie, 2011). High levels of insolation together with temperatures greater than 30°C during the sugar accumulation period were found to promote berry growth (Jones and Davis, 2000), while limited sunlight during veraison delayed grape ripening (Keller et al., 1998). Results from Bergqvist et al. (2001) suggested that the effects of light on fruit composition are heavily dependent upon the extent to which berry temperature is elevated as a result of increased PAR.

Vine water status can also have an important effect on sugar accumulation rates due to its effect on photosynthesis (Zufferey et al., 2000), shoot growth (Pellegrino et al., 2005), berry weight (Ojeda et al., 2001), berry water budget (Greenspan et al., 1994), and carbohydrate partitioning (Dry and Loveys, 1998). Water deficits can also increase berry sugar concentrations when moderate, but decrease berry sugar concentrations when severe (Peyrot des Gachons et al., 2005; van Leeuwen et al., 2009). Individual cultivars manage their water status differently in response to changes in climatic conditions (Schultz, 2003; Domec and Johnson, 2012). The effects of these and other climatic variables on sugar accumulation traits have been studied, but not extensively quantified for a wide variety of grape genotypes under comparable conditions.

Sugar accumulation traits can also be affected indirectly by other factors, such as phenology and berry weight, and their responses to changes in climate. There is significant genotypic variation in the phenology of different cultivars and in their phenotypic plasticity in response to antecedent temperatures conditions (Parker et al., 2011, 2013). In turn, within a given year, such differences in phenology will have an important effect on the climate conditions (temperature, PAR, soil water deficits) experienced by each cultivar during different stages of its development (van Leeuwen and Darriet, 2016).

It is difficult with this type of research to find data from multiple cultivars grown in the same climate in a sound experimental layout, such as with a randomized block design. The cultivar repositories that exist to date often have not been planted with replicates, making it impossible to study genotype × environment interactions (Destrac Irvine and van Leeuwen, 2016). And generally, such genotypic and phenotypic data is available for only a limited set of widely grown varieties (Wolkovich et al., 2018). The VitAdapt experimental vineyard is unique in that it allows for collection of data with replicates, allowing for statistically robust analysis across a wide range of varieties (Destrac Irvine and van Leeuwen, 2016).

The purpose of this study is to describe key sugar accumulation traits and characterize their plasticity in response to seasonal variation in climatic and other variables for 36 different grapevine cultivars using data collected over 7 years (2012–2018) from an experimental vineyard in Bordeaux France. More specifically, the four objectives of this study are to: (i) fit a sigmoidal model to collected data and quantify key sugar accumulation traits; (ii) characterize and classify the 36 cultivars based on these sugar accumulation traits; (iii) determine the relative effect of genetic versus various environmental controls on these sugar accumulation traits; and (iv) evaluate how much of the variability in the plasticity of these sugar accumulation traits are affected by different independent variables.

## Materials and Methods

### Experimental Setting

Data for this study was collected in the VitAdapt experimental vineyard at Domaine de la Grande Ferrade of the INRAE (Institut national de recherche pour l’agriculture, l’alimentation et l’environnement) research center in Bordeaux, France (44°47′23.8″N, 0°34′39.3″W) (Destrac Irvine and van Leeuwen, 2016). The VitAdapt vineyard was planted with 48 *V. Vinifera* (L.) and four hybrid cultivars in 2009, with the purpose of studying the response of these varieties to climate change in Bordeaux. The cultivars have been phenotyped each year starting in 2012 for many traits, including phenology (mid-budbreak, mid-flowering, and mid-veraison), grape composition during ripening, carbon isotope discrimination in berry juice sugars, and others. The cultivar names, origins, clones, and years of plantation are listed in Supplementary Table 1. The vineyard is located on a relatively homogeneous gravel soil in the Pessac-Léognan appellation. Soil physical and chemical properties are presented in Supplementary Table 2.

The 0.6 ha vineyard has 46 rows with five buffer vines at each end of each row that are not included in the study. The vineyard was laid out using a randomized block design with four blocks for each cultivar and each block consisting of two parallel rows of five grapevines each. All grapevine clones were grafted on Sélection Oppenheim 4 (SO4) rootstock. Irrigation was necessary at plantation in 2009 and in July of the same year. Once the rooting system was established, however, the vines have been dry-farmed. The vines are Guyot pruned and trained with a vertical shoot positioned trellis. The vines were estimated to have 2.0–2.4 m^2^ of canopy leaf area per meter of row. The vineyard was managed according to good agricultural practices.

### Phenology Observations

Phenological stages of all cultivars were tracked following the Biologische Bundesanstalt, Bundessortenamt und CHemische Industrie (BBCH) scale for monocots and dicots as described by Hack et al. (1992). Dates of mid-flowering (BBCH 65) mid-veraison (BBCH 85) were recorded for each cultivar through field observations. These are denoted as:

t_flo_ = time (DOY) of mid-flowering.
t_ver_ = time (DOY) of mid-veraison.

During each phenological stage, observations were carried out on Mondays, Wednesdays, and Fridays for each cultivar and replicated until the mid-point of that stage was identified. For missing data (7.7% of total), replacement values were obtained by averaging values from the other blocks for the same cultivar in the same year.

### Climate and Water Stress Indicators

Climatic data were recorded by a weather station situated approximately 100 m from the experimental vineyard. The station is part of the CIMEL automated DEMETER network, and the data is obtained from the INRAE (Institut national de recherche pour l’agriculture, l’alimentation et l’environnement) Climatik meteorological database for the Villenave d’Ornon (la Grande-Ferrade) location.

To account for the differences in phenology between the cultivars, the observed dates of mid-flowering and mid-veraison are used for each replicate (cultivar × year × block) to calculate the various climate statistics used in this analysis. The following climate variables were used as input to the analysis of variance (ANOVA):

T_f–v_ = average air temperature (°C) between mid-flowering (t_flo_) and mid-veraison (t_ver_).
T_v–95_ = average air temperature (°C) between t_ver_ and time (DOY) of 95% maximum sugar content/concentration (t_95_).
PAR_f–v_ = average photosynthetic active radiation (J cm^–2^) between t_flo_ and t_ver_.
PAR_v–95_ = average photosynthetic active radiation (J cm^–2^) between t_ver_ and t_95_.
RR_f–v_ = total rainfall (mm) between t_flo_ and t_ver_.

In the absence of any direct measurements of soil water deficit or vine water status prior to mid-veraison, total rainfall between mid-flowering and mid-veraison is considered a good surrogate. After veraison, carbon isotope discrimination in berry juice sugar is well correlated to plant water status during the period of sugar accumulation (Gaudillère et al., 2002). Although intrinsically δ^13^C is a measure of water use efficiency (WUE), is also a very good proxy for vine water status (van Leeuwen et al., 2009; Santesteban et al., 2015) δ^13^C measurements were performed each year prior to harvest for every cultivar and every block. For missing δ^13^C data (about 3%), to allow balanced statistical analysis, replacement values were obtained by averaging values from the other blocks for the same cultivar in the same year.

### Berry Components

The berry sugar concentration and content per berry and their respective rates of accumulation over the course of the season are the key traits being evaluated in this study across the different cultivars. The size of berries (as measured by weight) are also important factors influencing this development. Berries were analyzed during the 2012–2018 seasons starting at or a few days after mid-veraison. Approximately five healthy and representative berries per vine were harvested, adding to a total of 50 berries per plot. Berries were selected from different positions on the bunch. Berries were picked by hand and collected in bags containing a vertical filter (BagFilter^®^ 400 ml, Interscience, Saint-Nom-la-Bretèche, France). Each plot was sampled weekly in the morning between 08:00 and 10:30 a.m. Berries were counted and weighted collectively.

The juice of the berries was extracted using a crusher (BagMixer^®^ 400 W, Interscience, Saint-Nom-la-Bretèche, France) and collected in 50 ml tubes. The tubes were centrifuged at 20,089 × *g* for 10 min (Sigma 6K15, SIGMA Laborzentrifugen GmbH, Osterode am Harz, Germany). The juice was then analyzed by using a WineScan^TM^ Auto based on Fourier Transform Infrared Spectroscopy (FTIR; FOSS Analytical, Hillerød, Denmark) (Destrac Irvine et al., 2015). Values of reducing sugars produced by the WineScan^TM^ Auto were validated with a digital refractometer and were found to be similar (*P* < 0.05). Sugar content was calculated from berry weight and sugar concentration.

### Statistical Analysis

#### Sugar Accumulation Curve Fitting

Values for reducing sugar were fitted with a non-linear model. Both sugar content and concentration in berries versus time follow a sigmoid curve and were well fitted by a 3-parameter logistic function (Eq. 1; Triboï et al., 2003) as given by:

(1) $$S\left(t\right)=\frac{S_{max}}{1+0.05\cdot e^{\left(-4\cdot r\cdot \left(\frac{t-t_{95}}{S_{max}}\right)\right)}}$$

where, S_max_ = the estimated maximum content or concentration of reduced sugars, *t* = day of year (DOY), t_95_ = DOY when 95% of maximum was accumulated, and *r* represents the estimated maximum rate of accumulation defined as the derivative at the point of inflection. With this model the amount of sugar (as either content, or concentration) at mid-veraison (S_ver_) and at 95% sugar accumulation (S_95_) can be iteratively calculated. Each block for a cultivar was modeled separately in order that ANOVA could be performed. The modeling was implemented with the data expressed both in concentration (g L^–1^) and content (mg berry^–1^). The following traits were extracted from the model:

t_95–conc_ = day of year (DOY) when sugar concentration reached 95% of maximum.
t_95–cont_ = day of year (DOY) when sugar content reached 95% of maximum.
S_ver–conc_ = sugar concentration (g L^–1^) at t_ver_.
S_ver–cont_ = sugar content (mg berry^–1^) at t_ver_.
S_95–conc_ = sugar concentration (g L^–1^) at t_95–conc_.
S_95–cont_ = sugar content (mg berry^–1^) at t_95–cont_.
r_conc_ = maximum rate of sugar accumulation concentration (g L^–1^ day^–1^).
r_cont_ = maximum rate of sugar accumulation as content (mg berry^–1^ day^–1^).
Dur_conc_ = number of days between t_ver_ and t_95–conc_.
Dur_cont_ = number of days between t_ver_ and t_95–cont_.
BW_v_ = berry weight (g) at t_ver_.

Data were statistically analyzed using the open source software R (R Core Team, 2017) within the integrated development environment RStudio. The nonlinear model was fitted using the R function *nls*. The performance of the model for each cultivar was evaluated by calculating the coefficient of determination (*r*^2^) and the root-mean-square error (RMSE). Assumptions of normality and equal variance were respectively checked by quantile plots and plotting standardized model residuals against fitted values, respectively. The standardized residual plots of the models were found to be homoscedastic. Graphs and tables were produced with RStudio and Microsoft Office Excel 2010.

#### Hierarchical Cluster Analysis

A clustering was performed to find structures within the gene pool (represented by the cultivars) based on key sugar accumulation traits. The clustering in Figure 4 was made using the Euclidean distance measure. The six identified clusters closely resembled that of k-means clustering through minimization of within-cluster sum of squares. The clustering was based on t_ver_, S_95–conc_, and Dur_cont_, which are key traits from both winemaking and physiological perspectives.

#### Correlation, ANOVA, and Multiple Regression Analysis

##### Correlation and ANOVA

Bravais-Pearson correlation coefficients (*r*) were calculated between traits of all cultivars together. ANOVA was used to determine the effect of year, cultivar, and their interaction on the modeled sugar accumulation traits. The sum-of-squares was used to determine their contribution to the explained variance in the traits. Type III ANOVA was used to quantify more specific genetic and environmental controls on the traits. Second-order interactions, albeit sometimes significant, were excluded from the analysis since they accounted for no more than 2.8% (no more than 16.1% together) of the total variance for each sugar accumulation trait. Multicollinearity was tested through calculation of variance inflation factors (VIF), which all remained less than six and were deemed acceptable given the large dataset. The *relaimpo* package in R (Grömping, 2006) was used to determine the contribution of regressors in explaining the variance of the traits. Tukey’s HSD test was used for *post hoc* pairwise comparisons across cultivar means for the traits (Supplementary Table 3).

##### Multiple linear regression analysis

Multiple linear regression analysis was performed for each cultivar between the dependent traits in Dur_cont_, S_95–cont_, and S_95–conc_ against various independent genetic and environmental variables. The optimum set of variables in each model was determined through an “all-possible combinations” regression method. The model selected for each individual cultivar had the highest possible adjusted *r*^2^, while model coefficients were significant (*P* < 0.05) and showed low multicollinearity (VIF < 2).

## Results

### Climatic Conditions Experienced by Grape Cultivars

**FIGURE 1:**
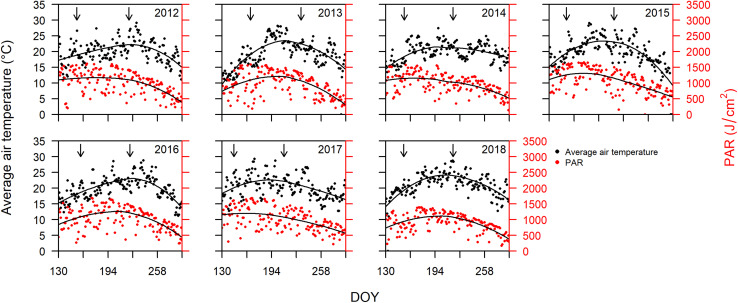
Average daily air temperature (°C) on primary vertical axis, and photosynthetically active radiation (PAR; J cm^–2^) on the secondary vertical axis, plotted versus day of year (DOY) between DOY 130 and 290 for years 2012 through 2018. Arrows indicate dates of mid-flowering and mid-veraison averaged over all cultivars for each year. The solid lines represent LOESS (locally estimated scatterplot smoothing) fitted to the data for temperature and PAR.

Bordeaux is classified as Cfb (warm temperate, fully humid, warm summers) according to the Köppen and Geiger climate classification (Beck et al., 2018). Cool and wet winters and springs, followed by warm and dry summers are responsible for some of the best wine vintages in Bordeaux (Baciocco, 2014). The average daily air temperature and PAR from DOY 130 to 290 for years 2012 to 2018 are plotted in Figure 1. PAR is closely related to temperature, but with slightly different trends over the progression of the different seasons. The peak in temperatures always occurs after the peak in PAR (Figure 1).

Since sugar accumulation in grape berries mainly occurs from veraison to maturity (Coombe and McCarthy, 2000), we further analyzed the climate variables for the periods prior and post to mid-veraison. The average temperature from mid-flowering to mid-veraison (T_f–v_) was higher than that from mid-veraison to maturity in four out of the 7 years studied (Table 1). The average PAR from mid-flowering to mid-veraison (PAR_f–v_) was higher in all 7 years compared to those from mid-veraison to maturity. In addition to temperature, the moderate to severe soil water deficits experienced in Bordeaux during berry ripening are also associated with high quality vintages (van Leeuwen et al., 2009). The total rainfall from flowering to veraison was higher than those from veraison to maturity in 6 out the 7 years.

**TABLE 1:** Average air temperatures, average PAR, and total rainfall over the flowering to veraison and the veraison to maturity periods (dates averaged across all cultivars) for 2012 to 2018.

	Average air temperature (°C)		Average PAR (J cm^–2^)		Total rainfall (mm)	
Year	Mid-flowering – veraison	Veraison – maturity	Flowering – veraison	Veraison – maturity	Flowering – veraison	Veraison – maturity
2012	20.3	22.0	1133.1	954.7	120	48
2013	22.0	19.4	1155.6	827.9	214	48
2014	21.4	20.7	1153.6	943.8	140	86
2015	22.5	21.3	1285.9	1013.3	46	111
2016	20.9	22.3	1192.2	1051.1	90	64
2017	22.1	21.2	1162.5	977.9	168	50
2018	22.9	23.1	1065.4	985.5	106	76

There is considerable variability in the observed phenology across the 36 cultivars (Destrac Irvine and van Leeuwen, 2016). This phenological variability may affect the climatic conditions experienced by each cultivar within a given year. An ANOVA was conducted to assess the relative contributions of cultivar and year on the exact climatic conditions experienced by each cultivar over different development stages (Table 2). As expected, year explained a large amount of the variability (20.4–94.4%) in the climate conditions experienced by each cultivar. Interestingly, the T_f–v_, T_v–95_, and PAR_v–95_ were also largely influenced (13.1–25.3%) by cultivar-specific phenology (Table 2). In addition, the BW_v_ and δ^13^C, two proxies of climatic conditions (see section “Materials and methods”), were also analyzed. The year effect was responsible for 63.5% of the variability in δ^13^C, while BW_V_ was mainly influenced by cultivar (57.8%). These results highlight the necessity to distinguish between antecedent and concurrent factors when analyzing the potential linkages between independent variables and sugar accumulation traits.

**TABLE 2:** ANOVA analysis illustrating the effect of cultivar, year, and their interaction on pre- and post-veraison climatic variables, berry size, and δ^13^C.

	Contribution of variance components (%)							
Source	Degrees of freedom	RR_f–v_	PAR_f–v_	T_f–v_	PAR_v–95_	T_v–95_	BW_v_	δ^13^C
Cultivar	35	2.1	1.1	14.7	25.3	13.1	57.8	7.9
Year	6	87.4	94.4	85.2	35.1	54.0	20.4	63.5
Cultivar × year	210	8.7	4.5	0.1	20.3	15.1	9.1	6.1*
Residuals	756	1.8	0.0	0.0	19.3	17.8	12.7	22.4
Total variance explained		98.2	100.0	100.0	80.7	82.2	87.3	77.6

*ns, not significant.

### Modeling Sugar Accumulation

**FIGURE 2:**
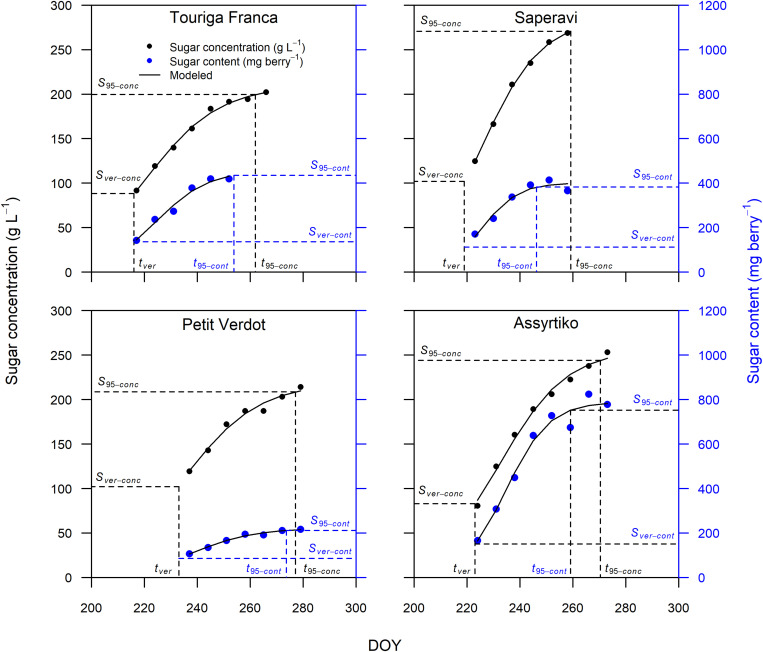
Sugar accumulation data and fitted curves for Touriga Franca, Saperavi, Petit Verdot, and Assyrtiko expressed in both concentration (in black) and content (in blue) for 2016 (block 1). Vertical dashed lines identify t_ver_ and t_9__5_ concentration, or content. Horizontal dashed lines identify the corresponding sugar concentration (on primary vertical axis), or content (on secondary vertical axis).

**FIGURE 3:**
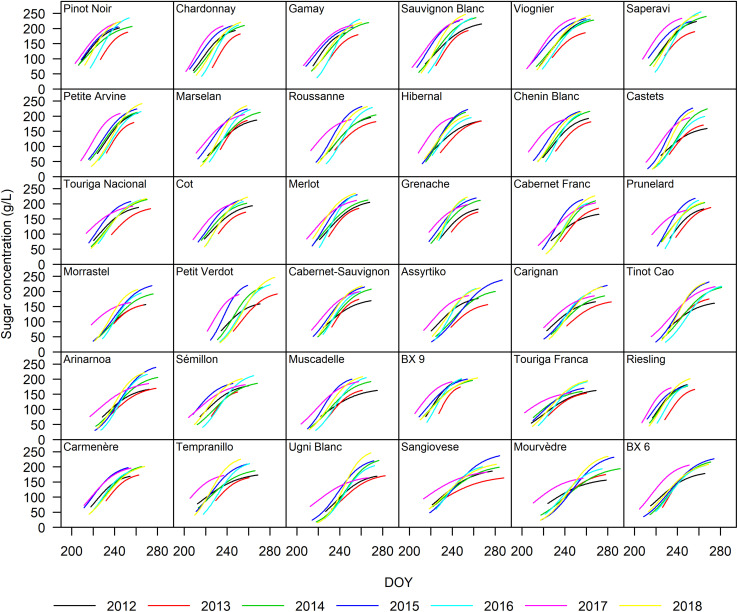
Sugar accumulation dynamics of the 36 grape cultivars included in this study from 2012 to 2018. The curves represent a single year and were drawn from traits averaged over the four blocks.

The sigmoidal model was applied as described in the section “Materials and Methods” to each single replicate (year × cultivar × block) providing a statistically strong fit. Figure 2 presents the curve fits for four cultivars showing the sugar accumulation traits expressed both as concentration and content. The cultivars Touriga Franca and Saperavi consistently attain the lowest and highest berry sugar concentrations in this dataset, respectively. Whereas the cultivars Petit Verdot and Assyrtiko accumulate the lowest and highest berry sugar contents, respectively. The *r*^2^ for each cultivar (averaged over blocks) was between 0.96–0.99 and 0.92–0.98 when expressed in concentration and content, respectively (Supplementary Table 4). RMSE for the curve fits for concentrations and content across all cultivars were between 3.30 and 5.56 g L^–1^ and between 9.73 and 29.49 mg berry^–1^, respectively. The model performed well over a large range of cultivars with different sugar accumulation dynamics under different environmental conditions. Figure 3 illustrates the variation in sugar accumulation curves for each cultivar (the same curves in mg berry^–1^ can be found in Supplementary Figure 1).

### Characterizing Cultivars by Accumulation Traits

#### Cluster Analysis

**FIGURE 4:**
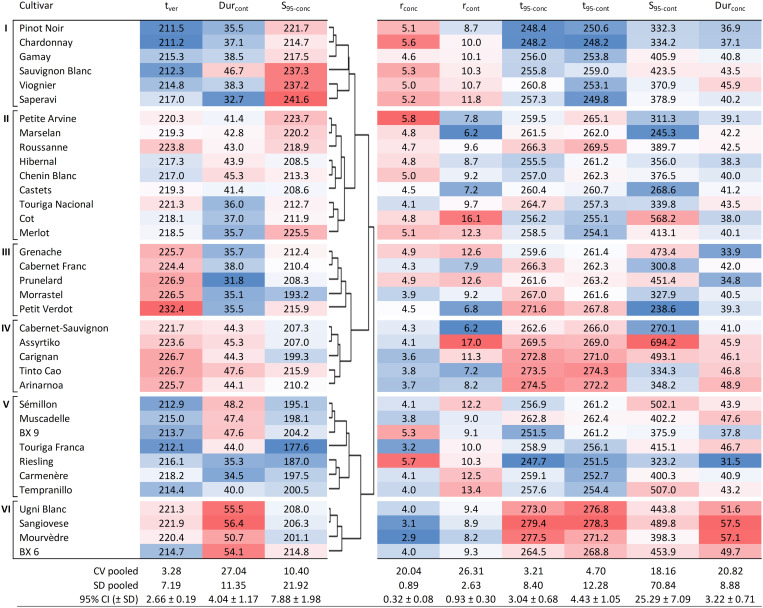
Hierarchical cluster analysis based on t_ver_, Dur_cont_, and S_95–conc_, showing six groups for the 36 cultivars together with other sugar accumulation traits for reference. Values shown are averages of each trait for each cultivar (over 7 years and four blocks, *n* = 28). Blue cells are increasingly lower values and red cells are increasingly higher values within the range observed for each of the traits. DOY, day of the year; CV pooled, pooled coefficient of variation; SD pooled, pooled standard deviation; 95% CI, average 95% confidence interval with standard deviation.

Figure 4 presents the clustering analysis of the 36 cultivars based on t_ver_, S_95–conc_, Dur_cont_, showing the resulting six clusters, along with all the other sugar accumulation traits for reference (except for S_ver–conc_ and S_ver–cont_ which can be found in Supplementary Table 3). The timing of mid-veraison was selected for the clustering analysis as it can indirectly affect sugar accumulation. It drives the start of ripening and may be a factor in determining both the length of time available for ripening and the climatic conditions that the vine and berries will experience during ripening (Table 2). Sugar concentration at 95% of maximum was selected as it determines the potential alcohol content of the wine and is of great interest to winemakers. The duration of the sugar accumulation period is of interest to growers, together with t_ver_, as it will determine in which part of the season the grapes will ripen. It may also be a concern with regard to achieving concurrent phenolic maturity of the grapes (Deloire et al., 2005). Expressed as content, this is the duration of active sugar loading to the berries and excludes the separate mechanism of sugar concentration caused by dehydration after loading has ceased. The main characteristics of the six clusters are described below.

Cluster I is characterized by cultivars that go through mid-veraison relatively early (t_ver_ < 217.0), ripen fast (Dur_cont_ < 38.5 days, with the exception of Sauvignon Blanc with 46.7 days) and show rather high concentrations of sugar at ripeness (214.7 < S_95–conc_ < 241.6 g L^–1^).

Cluster II is a mix of cultivars that go through mid-veraison on average more than 5 days later than those in Cluster I. S_95–conc_ are generally slightly lower and Dur_cont_ is notably lower for Touriga Nacional, Cot, and Merlot.

Cluster III cultivars go through mid-veraison later (t_ver_ > 224.4), but with short sugar accumulation periods (Dur_cont_ < 38.0 days). In this cluster, Petit Verdot is notably the latest grape cultivar to go through mid-veraison, but not the latest to reach a plateau in sugar concentration (t_95–conc_) due to quick ripening.

Cluster IV cultivars have similar ripening dynamics to Cluster II. The major difference between Clusters II and IV is that the latter display only lower levels of sugar concentration and need more time to load sugars.

Cluster V represents cultivars that go through mid-veraison early (t_ver_ < 218.2) and end up with low concentrations of sugars (S_95–conc_ < 204.2 g L^–1^) at ripeness. Touriga Franca is very typical for this behavior.

Cluster VI cultivars were not the latest to go through mid-veraison, but ripened the slowest and therefore have the longest ripening period (Dur_cont_ > 50.7 days).

In general, the coefficients of variation were slightly higher for the sugar accumulation traits expressed in content compared to those expressed in concentration. Although the coefficients of variation for Dur_con__t_ and Dur_con__c_ were higher than those for S_95–conc_ and S_95–cont_, both indicate the presence of some plasticity (Figure 4).

#### Correlation Analysis

Correlations among the different sugar accumulation traits of the different cultivars were also evaluated to identify inter-relationships. Table 3 presents the Bravais-Pearson correlation coefficients (*r*) between all sugar accumulation traits, time of mid-veraison, and berry weight at mid-veraison for all replicates (cultivar × year × block) considered together. Correlations shown in bold had *r* > 0.71, which corresponds to the *r*^2^ > 0.5, meaning more than 50% of the variation in one variable explains the variation in the other variable.

**TABLE 3:** Bravais-Pearson coefficients (*r*) for correlation analysis performed between all sugar accumulation traits, t_ver_ and BW_v_ for all replicates (cultivar × year × block) considered together.

	t_ver_	r_conc_	r_cont_	S_95–conc_	S_95–cont_	t_95–conc_	t_95–cont_	Dur_conc_	Dur_cont_	BW_v_
t_ver_	–	ns	–0.11	–0.31	–0.21	0.50	0.47	–0.29	–0.16	–0.11
r_conc_	–	–	0.27	0.24	–0.16	–0.62	–0.33	−**0.73**	–0.40	–0.29
r_cont_	–	–	–	0.17	0.58	–0.22	–0.48	–0.16	–0.47	0.57
S_95–conc_	–	–	–	–	0.16	0.06	–0.21	0.33	ns	–0.19
S_95–cont_	–	–	–	–	–	0.10	0.17	0.29	0.33	**0.92**
t_95–conc_	–	–	–	–	–	–	0.59	0.68	0.32	0.11
t_95–cont_	–	–	–	–	–	–	–	0.26	**0.80**	0.16
Dur_conc_	–	–	–	–	–	–	–	–	0.49	0.21
Dur_cont_	–	–	–	–	–	–	–	–	–	0.26
BW_v_	–	–	–	–	–	–	–	–	–	–

*The correlations shown in bold had r > 0.71, or r^2^ > 0.50.*

In general, the rate of maximum increase in sugar concentration (r_conc_) was negatively correlated to both the time of 95% maximum sugar concentration (t_95__–__conc_) and the duration to that point starting at mid-veraison (Dur_conc_), with the latter two also being positively correlated. Likewise, the maximum berry sugar loading rate (r_cont_) was negatively correlated to both time of 95% maximum sugar content (t_95__–__cont_) and the duration to that point starting at mid-veraison (Dur_cont_), with the latter two also being positively correlated. Berry weight at mid-veraison (BW_v_) is strongly and positively correlated to sugar content at 95% of maximum (S_95__–__cont_).

Because all cultivars are considered together, the relationships between traits described in the above analysis are only general, with potentially much different, or possibly no such relationships existing for individual cultivars. Correlation analysis between these traits across years was also done separately for each cultivar (not shown) and found individual cultivars followed similar trends as the larger group, suggesting a consistency in these behaviors across individual cultivars, albeit with different slopes and intercepts.

### Climate Versus Genetic Effects on Accumulation Traits

To understand the relative effect of cultivar genetics versus climate, Table 4 presents an ANOVA showing the relative contribution of variance in sugar accumulation traits and t_ver_ associated with cultivar, year, cultivar × year interaction, and residuals, together with the total variance explained. Year, cultivar, and their interaction were highly significant (*P* < 0.001) and explained much of the variance of most of the traits. For the key traits t_ver_ and S_95–conc_, year explained around half of the variance, the latter showing a greater cultivar × year interaction effect. Dur_cont_ had relatively more variance explained by the cultivar and with larger cultivar × year interaction and residuals, although the overall variance explained by the model was less than the others at 59%. Much of the variation in S_95–cont_ is explained by cultivar, this indicates that differences in berry weight are strongly genotype-dependent. The remaining traits generally had more variance explained by the cultivar, with the rest explained relatively evenly across the other factors. The relative contribution of the block effect to the total variance of the various traits was low at between 0.1 and 0.8%.

**TABLE 4:** Analysis of variance showing relative contribution of variance in observed sugar accumulation-related traits associated with cultivar, year, cultivar × year interaction, block, and residuals, together with total variance explained.

	Contribution of variance components (%)									
Source	Degrees of freedom	t_ver_	Dur_cont_	S_95–conc_	r_conc_	r_cont_	t_95–conc_	t_95–cont_	S_95–cont_	Dur_conc_
Cultivar	35	35.5	24.4	27.0	40.2	47.0	48.2	29.3	64.0	29.4
Year	6	57.6	9.8	45.2	17.4	5.7	14.9	17.0	13.9	26.7
Cultivar × year	210	4.0	24.2	18.6	25.3	20.8	21.0	21.0	9.6	23.7
Block	3	0.2	0.5	0.2	0.1	0.8	0.2	0.2	0.7	0.3
Residuals	753	2.8	41.2	9.1	16.9	25.8	15.7	32.5	11.8	19.9
Total variance explained		97.2	58.8	90.9	83.1	74.2	84.3	67.5	88.2	80.1

### Plasticity of Accumulation Traits

#### General Effects

Type III ANOVA was performed to test the main effects of climate related variables, t_ver_ and BW_v_ on the different sugar accumulation traits (Table 5). Overall, the total variation of the content-based sugar accumulation traits was better explained (48–77%) than the total variation for the concentration-based traits (28–44%). Rainfall prior to mid-veraison explains 44.1% of the variance in sugar concentration at maturity (S_95–conc_). Average post mid-veraison PAR explained more of the variance than post mid-veraison temperature for four of the eight sugar accumulation traits and it explained a large part of the variation in three of the four content-based sugar accumulation traits. Sugar content per berry is strongly affected by berry weight at mid-veraison.

**TABLE 5:** Analysis of variance as performed by type III ANOVA showing the amount of variance in the eight sugar accumulation traits explained by different environmental variables for all cultivars considered together.

	Relative contribution of variance components (%)							
Source	Dur_cont_	S_95–conc_	r_conc_	r_cont_	t_95–conc_	t_95–cont_	S_95–cont_	Dur_conc_
t_ver_	22.7	ns	12.4	7.6	43.5	ns	ns	27.2
BW_v_	6.0	6.6	21.7	31.4	3.0	3.1	88.7	7.6
T_f–v_	7.7	11.9	ns	7.5	ns	8.1	1.6	ns
PAR_f–v_	ns	7.2	2.7	1.0	7.4	ns	0.9	8.3
RR_f–v_	8.4	44.1	7.3	0.7	12.3	3.6	5.8	32.4
T_v–95_	13.0	ns	31.2	ns	30.7	20.9	ns	22.8
PAR_v–95_	40.3	13.6	22.8	51.9	ns	59.4	ns	ns
δ^13^C	1.9	16.5	2.0	ns	3.0	4.8	2.9	1.8
*r*^2^ model	71.8	36.8	28.0	47.5	44.3	77.4	70.3	32.2

*ANOVA run with n = 1008 individual observations. ns, not significant.*

With all cultivar data considered together, this type of analysis identifies larger general trends, but can be blurred by the differential behavior of the 36 individual cultivars. Performing this analysis on a per cultivar basis will improve understanding of the dynamics.

#### Cultivar-Specific Effects

The effects of the same climatic factors, t_ver_ and BW_v_ included in Table 5 are evaluated to quantify their contribution to the observed variation in the key sugar accumulation traits of Dur_cont_, S_95–cont_, and S_95–conc_ for all the cultivars individually. Dur_cont_ is of interest to growers, together with t_ver_, as it will determine in which part of the season the grapes will ripen. It may also be of interest with regards to achieving concurrent phenolic maturity of the grapes. Expressed as content, this is the duration of active sugar loading to the berries and excludes the separate mechanism of sugar concentration caused by dehydration after loading has ceased. S_95–cont_ is of physiologic interest as it is the ultimate amount of sugar loaded into the berry, and S_95–conc_ is of interest to winemakers as it drives the potential alcohol content, which is important in the final sensory attributes of the wine.

Multiple linear regression analysis was performed for each cultivar on the above dependent traits against the same set of independent variables in Table 5. On average the resulting models explained 69, 70, and 69% of the total variation in Dur_cont_, S_95–conc_, and S_95–cont_, respectively and are described in more detail below.

##### *Duration in content (Dur*_cont_*)*

**FIGURE 5:**
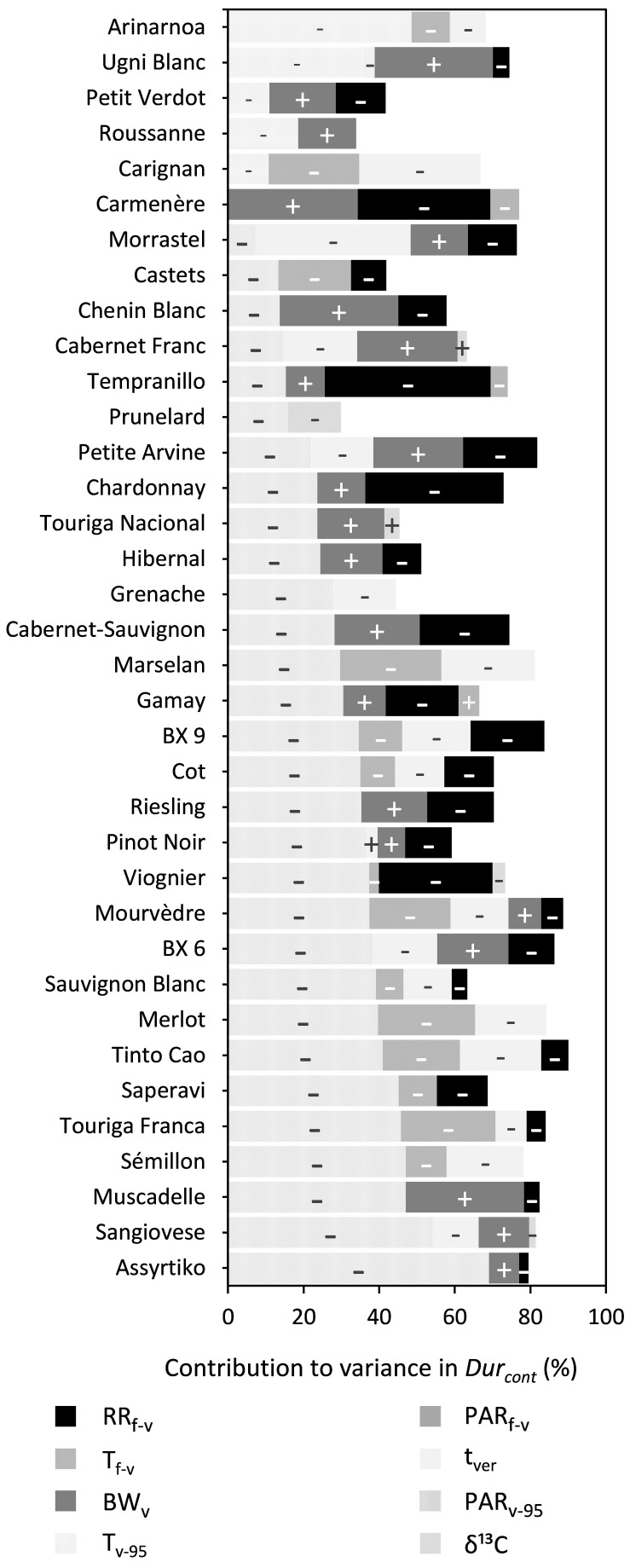
Stacked bar plots of the variance components determined by multiple linear regression for trait Dur_cont_. The cultivars are ordered based on the percentage explained by PAR_f–v_. The independent variables included were RR_f–v_, PAR_f–v_, T_f–v_, PAR_v–95_, T_v–95_, t_ver_, BW_v_, and δ^13^C. The – and + represent the directions in which coefficients of variables moved. Supplementary Table 6 provides a numeric form of this figure.

PAR_v–95_, RR_f–v_, BW_v_, t_ver_, and T_f–v_ were shown to significantly influence Dur_cont_ for 30, 25, 21, 19, and 14 out of the 36 cultivars, respectively, although the relative contribution of each was very different across the cultivars (Figure 5 and Supplementary Table 5). An increase in PAR_v–95_, RR_f–v_, T_f–v_, or t_ver_ reduced Dur_cont_, while an increase in BW_v_ increased Dur_cont_. Whereas PAR_v–95_ was important for most cultivars, it was replaced by T_v–95_ for the cultivars Arinarnoa, Ugni Blanc, Petit Verdot, Roussanne, and Carignan. During the mid-flowering to mid-veraison period, the effect of PAR_f–v_ was relatively small across all cultivars. During the mid-veraison to maturity period the effect of vines water status assessed with δ^13^C was also relatively small.

##### *95% maximum sugar content (S*_95–cont_*)*

**FIGURE 6:**
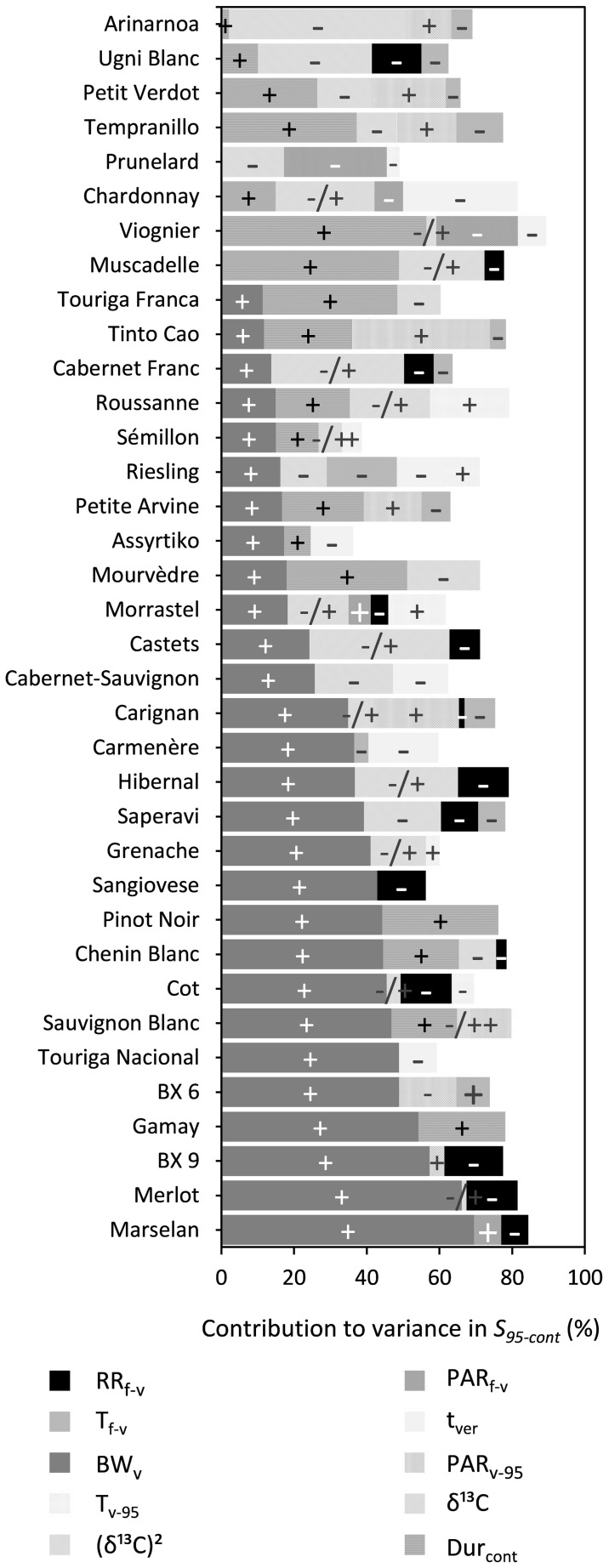
Stacked bar plots of the variance components as determined by multiple linear regression for the trait S_95–cont_. The cultivars are ordered based on the percentage explained by BW_v_. The independent variables included were RR_f–v_, PAR_f–v_, T_f–v_, PAR_v–95_, T_v–95_, t_ver_, BW_v_, and the trait Dur_cont_. δ^13^C was included as a linear term, whereas (δ^13^C)^2^ was included using a power term into the regression. The –, + and –/+ represent the directions in which coefficients of variables moved. Where –/+ was only designated to the variable (δ^13^C)^2^ because of its inherent quadratic character. Supplementary Table 7 provides a numeric form of this figure.

BW_v_, water deficit [δ^13^C or (δ^13^C)^2^], Dur_cont_, and RR_f–v_ were shown to significantly influence S_95–cont_ for 28, 25, 18, and 14 out of the 36 cultivars, respectively, while the effects of T_f–v_ and T_v–95_ were generally small for most cultivars (Figure 6 and Supplementary Table 6). An increase in BW_v_ or Dur_cont_ increased S_95–cont_, while increased water deficit after veraison (δ^13^C) and RR_f–v_ reduced S_95–cont_. Water deficit after veraison was included either as a linear or as a nonlinear function (δ^13^C)^2^ in the multiple regression. The effect of (δ^13^C)^2^ was quadratic, meaning that S_95–cont_ increased with water deficit until a maximum and then decreased, if water deficit continued to increase.

##### *95% maximum sugar concentration (S*_95–conc_*)*

**FIGURE 7:**
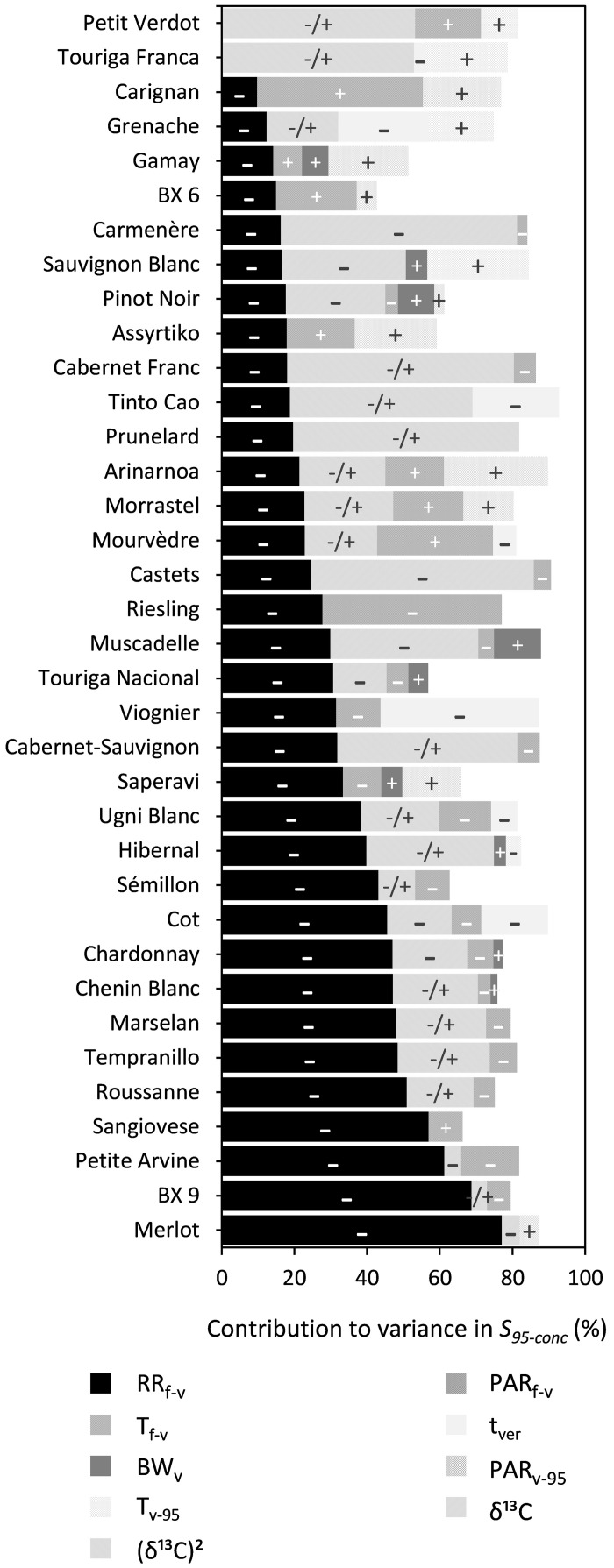
Stacked bar plots of the variance components as determined by multiple linear regression for the trait S_95–conc_. The cultivars are ordered based on the percentage explained by RR_f–v_. The independent variables included were: RR_f–v_, PAR_f–v_, T_f–v_, PAR_v–95_, T_v–95_, t_ver_, and BW_v_. δ^13^C was included as a linear term, whereas (δ^13^C)^2^ was included using a power term into the regression. The –, + and –/+ represent the directions in which coefficients of variables moved. Where –/+ was only designated to the variable (δ^13^C)^2^ because of its inherent quadratic character. Supplementary Table 8 provides a numeric form of this figure.

RR_f–v_, PAR_f–v_, and (δ^13^C)^2^ were shown to significantly influence S_95–conc_ for 34, 20, and 18 out of the 36 cultivars, respectively (Figure 7 and Supplementary Table 7). The effects of BW_v_ and t_ver_ were generally small and RR_f–v_ and PAR_f–v_ reduced S_95–conc_. The influence of (δ^13^C)^2^ was quadratic, meaning that S_95–conc_ reached an optimum at a certain level of water deficit.

## Discussion

Berry sugar accumulation data from 36 grapevine cultivars were collected between veraison and maturity over 7 years from a vineyard in Bordeaux. Sigmoid curves provided a strong statistical fit to the data and were used to obtain key sugar accumulation traits. The diversity of these traits were then described and the dynamics of the sugar accumulation rate, duration, and concentration/content at maturity traits across cultivars were studied. Other grapevine traits that can influence sugar accumulation traits, such as phenology, berry weight, and water deficit response, were also considered.

### Characterizing Cultivars

Clustering analysis was performed to assess similarities (or not) among cultivars across the 7 years of the study based on the date of mid-veraison (t_ver_), sugar concentration at 95% of maximum (S_95–conc_), and the duration between mid-veraison and the date of 95% sugar content (Dur_cont_). Although the physiological mechanisms driving them differ, these traits are of agronomic interest and the clustering (as presented in Figure 4) provides a useful categorization of cultivars for easy reference.

Correlation analysis between the different sugar accumulation traits of all cultivars considered together found important general relationships between the maximum rate of accumulation, the duration, and the date of maturity. Faster maximum accumulation rates were generally associated with both earlier maturity dates and shorter durations between mid-veraison and maturity. Individual cultivars followed similar trends as the larger group, suggesting some consistency in these behaviors across cultivars, albeit with different slopes and intercepts.

### Climate Versus Genetics

For each individual ripening trait, the results of this study give insight into how much their variation is driven by climate, cultivar, and climate × cultivar interaction.

Analysis of variance analysis of the variation in sugar accumulation traits, for all cultivars considered together, were well explained by cultivar, year, and their interaction, with total variance explained ranging between 59 and 97% across the different traits.

For the key traits of t_ver_ and S_95–conc_, year explained around half of their respective variances, while cultivar explained roughly one third. This suggests that climate was a strong driver of those traits, with genetic variation also being important. About 19% of variance in S_95–conc_ was explained by cultivar × year interaction, suggesting an additional contribution from phenotypic plasticity.

Compared to all other sugar accumulation traits, the ANOVA analysis found Dur_cont_ had the lowest amount of total variance explained (58.8%), suggesting other variables not included in the model had an effect. Of the total variance for this trait, little was explained by year, with both cultivar and cultivar × year interaction each explaining about 24%. The remaining traits generally had more variance explained by cultivar with the rest explained relatively evenly by other factors.

### Phenotypic Plasticity

Analysis of variance analysis of sugar accumulation traits, with all cultivars considered together, found that the total variation of the content-based sugar accumulation traits were better explained by the selected environmental variables (48–77%) than the total variation for the concentration-based traits (28–44%). This could be due to the additional effect of berry dehydration on the latter that may occur after sugar loading is otherwise complete.

The following subsections provide a detailed look at the relationships between specific variables and the sugar accumulation traits of individual cultivars.

#### Pre -and Post-veraison Effects of PAR and Temperature

For duration as content (Dur_cont_), PAR_v–95_ had a significant influence in 30 out of 36 cultivars and T_v–95_ had a significant influence on 5 of 36 cultivars, although to varying degrees in both cases. PAR_v–95_ and T_v–95_ were not found together in the same model of Dur_cont_ for a given cultivar for reasons of collinearity (*r* = 0.73, *P* < 0.05). This, however, does not imply that there were no effects of temperature on cultivars when T_v–95_ was not included in the regression. All cultivars ripened between DOY 190 and 290 in any given year. During this period, days are already getting shorter, thereby reducing the amount of received PAR, while temperature is at its peak and relatively stable for approximately 20 days after t_ver_ before starting to decrease more steeply in September. In this study, the variation in PAR for a given temperature is therefore higher at high temperatures. In a year with early phenology, the sugar accumulation period may coincide with both higher temperature and PAR. This may be important as it has been shown that increased light and temperature levels may increase the photosynthetic rate of plants (McIntyre et al., 1982). A reduction in Dur_cont_ is possible if more sugar was partitioned to the berries through an increased rate of photosynthesis. Conversely, with a late phenology, a cultivar may ripen under lower temperature and light conditions. This in turn reduces the rate of photosynthesis and theoretically increases the duration of the sugar accumulation period. The effects of PAR_f–v_ and T_f–v_ on S_95–conc_ were found to be consistently small. For Dur_cont_, the variables PAR_f–v_ and T_f–v_ were not found together in the same model for reasons of collinearity. Higher levels of PAR prior to mid-veraison, when days were longer, might have resulted in higher amounts of sugars accumulated. The 95% maximum sugar content trait (S_95–cont_) showed only sporadic relationships between either temperature, or PAR, whether before, or after mid-veraison.

#### Effects of the Timing of Mid-Veraison

The trait t_ver_ may have an indirect effect on sugar accumulation. It drives the start of ripening and may be a factor in determining both the length of time available for ripening and the temperatures that the vine and berries will experience during ripening. The veraison date is influenced by both cultivar genetics and environmental conditions prior to veraison, in particular temperature (Parker et al., 2011). This makes t_ver_ somewhat of a proxy for those conditions driving the plasticity of sugar accumulation traits.

In this study, the duration of the sugar accumulation as content (Dur_cont_) decreased with delayed t_ver_ in 19 out of 36 cultivars, although to varying degrees. Cultivars with delayed t_ver_ ripened in less favorable conditions, and may be eventually halted, resulting in lower sugar contents and shorter Dur_cont_. In the 2013 growing season, not only were sugar accumulation rates lower, but final sugar contents were also lower for all cultivars (Figure 3). Date of mid-veraison (t_ver_) only showed sporadic relationships with 95% maximum sugar content (S_95–cont_) and sugar concentration (S_95–conc_).

#### Pre-veraison Effects of Rainfall

Rainfall (RR_f–v_) prior to mid-veraison had significant relationships with Dur_cont_, S_95–cont_, and S_95–conc_ across 25, 14, and 34 of 36 cultivars, respectively. Any physiological response to rainfall prior to mid-veraison is most probably driven by its effect on soil water status. In the absence of any available soil water, or water potential measurements prior to veraison, rainfall provides a good surrogate. The variability associated with evapotranspiration (the other parameter besides rainfall important in determining soil water status) is accounted for by average temperature and PAR from mid-flowering to mid-veraison as already included in the model when significant.

Rainfall prior to veraison was found to decrease berry sugar concentrations. An increase in soil water content might have resulted in an increased water supply to the berries and caused a subsequent dilution of S_95–conc_. In line with the previous statement, rainfall between budburst and veraison was found to be strongly correlated with berry weight at veraison (*r* = 0.77, *P* < 0.05). Another concurrent dilution effect may have been caused by partial failure of fruit-set and expansion (referred to as *millerandage* and *coulure* in French, or *hens and chickens* in English) caused by rainfall prior to mid-veraison. These two conditions can bring about a reduction in the number of berries per bunch, while increasing the size of the remaining berries.

Regarding its effect on reducing Dur_cont_, the affect of rainfall prior to mid-veraison is more difficult to explain. Such rainfall may increase vine vigor and thereby the rate of sugar accumulation, which is generally related to Dur_cont_ across all cultivars (Table 3).

#### Effects of Berry Weight at Veraison

Berry weight at mid-veraison appeared to be an important driver of final sugar content (S_95–cont_) for almost all cultivars in this study. Houel et al. (2013) obtained the same results for a wide range of cultivars and reasoned that cell division and cell expansion after anthesis were the main drivers of berry size. Any water deficit during the period could restrict berry cell division and thereby its potential final size (Shellie, 2014). On the other hand, cultivars such as Petit Verdot and Viognier vary little in berry weight. These cultivars are known for their relatively small and stable berry size to begin with and for these cultivars, the length of the sugar accumulation period explains most of the variation in S_95–cont_. However, for 21 out of the 36 cultivars, increased berry weight also increased Dur_cont_. A plausible explanation would be that bigger berries hold more sugar, and it takes more time to fill them.

#### Effects of the Duration of the Sugar Accumulation Period

Dur_cont_ was included as a variable in the multiple regression analysis to evaluate the effect of the length of the sugar accumulation period on S_95–cont_ (Figure 6) and no significant relationships were found. For some cultivars S_95–cont_ was quite variable for a given Dur_cont_ (e.g., Merlot and Mourvèdre). For these cultivars it was the level of water deficit and berry weight at mid-veraison that were also major determinants of S_95–cont_.

#### Effects of Water Deficit (δ^13^C)

For the trait of 95% maximum sugar content (S_95–cont_) and 95% maximum sugar concentration (S_95–conc_) water status post-veraison as measured by δ^13^C had an important effect for 25 and 18 cultivars of 36, respectively. δ^13^C was only sporadically related to Dur_cont_ and to a small degree. For some cultivars, the effect of water deficits was found to be linear. This was generally the case for cultivars that either did not experience weak or severe water deficits in the seven seasons in this study. Otherwise, this relationship with δ^13^C was best characterized by a power function, with higher sugar content or concentration when water deficit was moderate, and lower sugar content or concentration when water deficit was either weak or strong. This finding is consistent with van Leeuwen et al. (2009). Comparison of δ^13^C is useful within but not across cultivars, because high sugar contents may be reached at different levels of water deficits. When water deficit is moderate, shoot growth is more reduced compared to photosynthesis (Pellegrino et al., 2005). Hence, more sugar is available for berry ripening. This effect, combined with smaller berries under water deficit (Ojeda et al., 2001), results in higher sugar concentrations. Also, water deficit after veraison can lead to berry shrinkage by dehydration when the sum of xylem efflux and berry transpiration exceed phloem influx (Keller, 2006). Severe water deficits decrease photosynthetic activity and thereby limit sugar accumulation in berries (Zufferey et al., 2000). A dilution effect may occur at low water deficits as berry growth may be faster than sugar accumulation (Santesteban and Royo, 2006). Also, at low water deficits the rate of sugar accumulation appears to decrease relative to moderate water deficit stress. This might be due to more partitioning of sugar to vegetative growth over berry ripening (Dry and Loveys, 1998). Additionally, the response of berry growth to water deficit can also depend on the crop load (Trégoat et al., 2002).

### Effect of Leaf Area to Yield Ratios on Traits

The effect of yield (and its components) on sugar accumulation traits has been well-reported (Sadras and McCarthy, 2007; Dai et al., 2011; van Leeuwen and Darriet, 2016). Yield data was not recorded for the first 6 years of measurements and therefore was not included in the analyses. The grapevine canopy leaf area was estimated to be between 2.0 and 2.4 m^2^ per meter of row. According to Kliewer and Dokoozlian (2005) maximum levels of total soluble solids and berry weight are attained when leaf to fruit ratios are higher than 0.8–1.2 m^2^ kg^–1^. In such case, the canopies would be able to support 1.7–3.0 kg of fruit per meter of row. Although not consistently measured, occasional bunch counts and bunch weight assessments indicate that crop loads were lower than these levels in the vast majority of years. To the extent that actual leaf area to fruit load ratios may have limited sugar accumulation, this could explain some of the variance (residuals) not explained by the models.

### Significance of Findings From the Perspective of Climate Change

A major concern in agriculture, and in particular in grape growing for wine production, is that crop yields and quality may be significantly affected by climate change (Fraga et al., 2012), speaking to the need for adaptational strategies. Crop genetic diversity is a valuable resource to exploit as an adaptation to a changing climate (Morales-Castilla et al., 2020) and planting cultivars that are better suited to a region’s changing climate would allow grape growers to maintain cultivation in their current location.

Although the great genetic variation within the *V. vinifera* species is a valuable resource for adaptation (Wolkovich et al., 2018), phenotyping of relevant traits across the wide range of cultivars has been limited. Most existing data have been collected in cultivar repositories, which have not been planted with replicates, making it impossible to separate environmental from genotypic variability (Destrac Irvine and van Leeuwen, 2016). This study allowed for the evaluation of key traits relevant to winemakers and researchers regarding sugar accumulation in the context of climate change. Using multiple regression analysis, the variation in key sugar accumulation traits can be largely explained by climate variables such as PAR, temperature, and water status (both before and after mid-veraison), and by physiological variables such as berry weight and date of mid-veraison. The extent to which these different variables affected sugar accumulation traits, however, varied across grape cultivars. More research is needed to unravel the exact mechanisms underlying the differential genotypic responses of traits to environmental variables. Adaptation to climate change cannot be based on temperature alone and crop responses cannot be generalized across genotypes, even within species.

Climate change induces excessively high sugar levels in grapes, resulting in wines with increased alcohol content (Duchène and Schneider, 2005). It also results in earlier ripening, moving the ripening period to a part of the season when high temperatures are not optimum for producing high quality wines (van Leeuwen et al., 2019). Phenotyping specific sugar-related ripening traits across a wide range of cultivars provides useful information to growers, when adaptation to climate change drives them to change cultivars.

In this study we focussed only on sugar accumulation traits. Sugar, however, is only one of many determinants for grape cultivar suitability in wine regions. Other important traits include, but are not limited to, WUE, photosynthetic capacity, yield, and berry composition (e.g. organic acids, aroma precursors, tannins, color, etc.). Future research should focus on characterizing these key traits and their interaction with the environment to ultimately select for grape cultivars under future climate regimes. Also, the results observed in this study are likely to depend on climate, soil, clone, and rootstock. So to complement the results from this study it would be very useful if experimental vineyards like VitAdapt were set up in other winegrowing regions with different soils, clones, and rootstocks and under different climatic conditions. Such initiatives are already underway, such as the GreffAdapt project in Bordeaux where 55 different rootstocks are tested (Marguerit et al., 2018), and the BritAdapt project in the United Kingdom with a similar experimental set up as that in VitAdapt. The common garden design of the VitAdapt experimental vineyard provides a prototype for such experimental designs and is worth being reproduced in other winegrowing areas around the world.

## Conclusion

A sigmoidal model was successfully fit to weekly berry sugar accumulation data and key sugar accumulation traits were quantified. Cultivars were then clustered and characterized according to these traits. The variations in sugar accumulation traits for all cultivars, when considered together, were explained well by cultivar, year, and their interaction, highlighting the relative roles of genetic variation, climate factors, and phenotypic plasticity. As seen extensively in the literature and in practice, this study confirmed that increasing temperature has a significant effect on grapevine phenology, leading to advanced harvest dates. The results of this study, however, also showed that sugar accumulation traits were affected by other factors both antecedent and concurrent to veraison. Although the specific physiological mechanisms by which these traits respond to environmental variables remain to be identified, results of this study provide useful information to inform grower selection and management of cultivars in vineyards affected by climate change. Determining suitability of grapevine cultivars for existing or new winegrowing areas cannot be based on a single factor, as is currently done in most studies. Moreover, the major factors driving grape ripening dynamics cannot be generalized across cultivars.

## Data Availability Statement

The raw data supporting the conclusions of this article will be made available by the authors, without undue reservation.

## Author Contributions

CL and ADI contributed to the conception and design of the study. ADI was in charge of data collection. BS organized the database. BS and ZD performed the statistical analysis. MG and BS wrote the first draft of the manuscript. CL and ZD wrote sections of the manuscript. All authors contributed to manuscript revision, read and approved the submitted version.

## Conflict of Interest

The authors declare that the research was conducted in the absence of any commercial or financial relationships that could be construed as a potential conflict of interest.

## References

[B1] Baciocco K. A. (2014). Climate and Bordeaux wine quality: identifying the key factors that differentiate vintages based on consensus rankings. *J. Wine Res.* 25 75–90. 10.1080/09571264.2014.888649

[B2] Beck H. E. Zimmermann N. E. McVicar T. R. Vergopolan N. Berg A. Wood E. F. (2018). Present and future Köppen-Geiger climate classification maps at 1-km resolution. *Sci. Data* 5:180214. 10.1038/sdata.2018.214 30375988 PMC6207062

[B3] Bergqvist J. Dokoozlian N. Ebisuda N. (2001). Sunlight exposure and temperature effects on berry growth and composition of cabernet sauvignon and grenache in the central san Joaquin Valley of California. *Am. J. Enol. Vitic.* 52 1–7.

[B4] Conde C. Silva P. Fontes N. Dias A. C. P. Tavares R. M. Sousa M. J. (2007). Biochemical changes throughout grape berry development and fruit and wine quality. *Food* 1 1–22.

[B5] Coombe B. G. (1987). Influence of temperature on composition and quality of grapes. *Acta Hortic.* 206 23–35. 10.17660/ActaHortic.1987.206.1

[B6] Coombe B. G. McCarthy M. G. (2000). Dynamics of grape berry growth and physiology of ripening. *Aust. J. Grape Wine Res.* 6 131–135. 10.1111/j.1755-0238.2000.tb00171.x

[B7] Dai Z. Vivin P. Robert T. Milin S. Li S. H. Génard M. (2009). Model-based analysis of sugar accumulation in response to source - sink ratio and water supply in grape (*Vitis vinifera*) berries. *Funct. Plant Biol.* 36 527–540. 10.1071/FP08284 32688667

[B8] Dai Z. W. Ollat N. Gomès E. Decroocq S. Tandonnet J.-P. Bordenave L. (2011). Ecophysiological, genetic, and molecular causes of variation in grape berry weight and composition: a review. *Am. J. Enol. Vitic.* 62 413–425. 10.5344/ajev.2011.10116

[B9] Deloire A. Kraeva E. Martin M. Hunter J. J. (2005). “Sugar loading and phenolic accumulation as affected by ripeness level of Syrah/R99 grapes,” in *Proceedings of the XIVth International GESCO Viticulture Congress*, Geisenheim, 574–580.

[B10] Destrac Irvine A. Flutre T. Renaud C. Morin E. Durand L. Delrot S. (2015). “The use of fourier transform infrared spectroscopy in phenotyping berries from the grapevine *Vitis vinifera* L,” in *Proceedings of the XIXth International Giesco Meeting*, Montpellier, 614–645.

[B11] Destrac Irvine A. van Leeuwen C. (2016). “The VitAdapt project: extensive phenotyping of a wide range of varieties in order to optimize the use of genetic diversity within the *Vitis vinifera* species as a tool for adaptation to a changing environment,” in *Proceedings of the Sustainable Grape and Wine Production in the Context of Climate Change*, Bordeaux, 165–171.

[B12] Domec J.-C. Johnson D. M. (2012). Does homeostasis or disturbance of homeostasis in minimum leaf water potential explain the isohydric versus anisohydric behavior of *Vitis vinifera* L. cultivars? *Tree Physiol.* 32 245–248. 10.1093/treephys/tps013 22427373

[B13] Dry P. R. Loveys B. R. (1998). Factors influencing grapevine vigour and the potential for control with partial rootzone drying. *Aust. J. Grape Wine Res.* 4 140–148. 10.1111/j.1755-0238.1998.tb00143.x

[B14] Duchène E. Schneider C. (2005). Grapevine and climatic changes: a glance at the situation in Alsace. *Agron. Sustain. Dev.* 25 93–99. 10.1051/agro:2004057

[B15] Fraga H. Malheiro A. C. Moutinho-Pereira J. Santos J. A. (2012). An overview of climate change impacts on European viticulture. *Food Energy Secur.* 1 94–110. 10.1002/fes3.14

[B16] Gaudillère J. van Leeuwen C. Ollat N. (2002). Carbon isotope composition of sugars in grapevine, an integrated indicator of vineyard water status. *J. Exp. Bot.* 53 757–763. 10.1093/jexbot/53.369.757 11886896

[B17] Godden P. Wilkes E. Johnson D. (2015). Trends in the composition of Australian wine 1984-2014: composition of Australian wine 1984-2014. *Aust. J. Grape Wine Res.* 21 741–753. 10.1111/ajgw.12195

[B18] Greenspan M. D. Shackel K. A. Matthews M. A. (1994). Developmental changes in the diurnal water budget of the grape berry exposed to water deficits. *Plant Cell Environ.* 17 811–820. 10.1111/j.1365-3040.1994.tb00175.x

[B19] Greer D. Weedon M. (2014). Temperature-dependent responses of the berry developmental processes of three grapevine (*Vitis vinifera*) cultivars. *N. Z. J. Crop Hortic. Sci.* 42 233–246. 10.1080/01140671.2014.894921

[B20] Grömping U. (2006). Relative importance for linear regression in *R*: the Package relaimpo. *J. Stat. Softw.* 17 1–27. 10.18637/jss.v017.i01

[B21] Hack H. Bleiholder H. Buhr L. Meier U. Schnock-Fricke U. Weber E. (1992). Einheitliche Codierung der phänologischen Entwicklungsstadien mono- und dikotyler Pflanzen. Erweiterte BBCH-Skala, Allgemein. *Nachr. Dtsch. Pflanzenschutzd.* 44 265–270.

[B22] Hannah L. Roehrdanz P. R. Ikegami M. Shepard A. V. Shaw M. R. Tabor G. (2013). Climate change, wine, and conservation. *Proc. Natl. Acad. Sci. U.S.A.* 110 6907–6912. 10.1073/pnas.1210127110 23569231 PMC3637704

[B23] Houel C. Martin-Magniette M.-L. Nicolas S. D. Lacombe T. Le Cunff L. Franck D. (2013). Genetic variability of berry size in the grapevine (*Vitis vinifera* L.). *Aust. J. Grape Wine Res.* 19 208–220. 10.1111/ajgw.12021

[B24] Intergovernmental Panel on Climate Change [IPCC] (2014). “Summary for policymakers,” in *Climate Change 2014: Impacts, Adaptation, and Vulnerability. Part A: Global and Sectoral Aspects. Contribution of Working Group II to the Fifth Assessment Report of the Intergovernmental Panel on Climate Change*, eds Field C. B. Barros V. R. Dokken D. J. Mach K. J. Mastrandrea M. D. Bilir T. E. (Cambridge: Cambridge University Press).

[B25] Jones G. V. Davis R. E. (2000). Climate influences on grapevine phenology, grape composition, and wine production and quality for Bordeaux, France. *Am. J. Enol. Vitic.* 51 249–261.

[B26] Keller M. (2006). Ripening grape berries remain hydraulically connected to the shoot. *J. Exp. Bot.* 57 2577–2587. 10.1093/jxb/erl020 16868045

[B27] Keller M. Arnink K. J. Hrazdina G. (1998). Interaction of nitrogen availability during bloom and light intensity during Veraison. I. Effects on grapevine growth, fruit development, and ripening. *Am. J. Enol. Vitic.* 49 333–340.

[B28] Keller M. Shrestha P. M. Hall G. E. Bondada B. R. Davenport J. R. (2016). Arrested sugar accumulation and altered organic acid metabolism in grape berries affected by berry shrivel syndrome. *Am. J. Enol. Vitic.* 67 398–406. 10.5344/ajev.2016.16048

[B29] Kliewer W. M. Dokoozlian N. K. (2005). Leaf area/crop weight ratios of grapevines: influence on fruit composition and wine quality. *Am. J. Enol. Vitic.* 56 170–181.

[B30] Lecat B. Amspacher W. Higgins L. Lindsay Ferrara A. McGarry Wolf M. (2019). “2 - Wine sector: definitions and nuances from global to country analysis—A comparison between Old World, New World, and emerging wine countries from 2005 to current,” in *Case Studies in the Wine Industry. A Volume in the Consumer Science and Strategic Marketing Series Food Science, Technology and Nutrition* eds C. Santini and A. Cavicchi (Amsterdam: Elsevier), 7–32. 10.1016/B978-0-08-100944-4.00002-1

[B31] Marguerit E. Lagalle L. Lafargue M. Tandonnet J.-P. Goutouly J.-P. Roques M. (2018). “Greffadapt: a relevant experimental vineyard to speed up grapevine rootstock selection,” in *Proceedings of the GBG 2018*, Bordeaux, 278.

[B32] McIntyre G. N. Lider L. A. Ferrari N. L. (1982). The chronological classification of grapevine Phenology. *Am. J. Enol. Vitic.* 33 80–85.

[B33] Morales-Castilla I. García de Cortázar-Atauri I. Cook B. I. Lacombe T. Parker A. van Leeuwen C. (2020). Diversity buffers winegrowing regions from climate change losses. *Proc. Natl. Acad. Sci. U.S.A.* 117 2864–2869. 10.1073/pnas.1906731117 31988113 PMC7022210

[B34] OIV (2018). *2019 Statistical Report on World Vitiviniculture.* Paris: International Organisation of Vine and Wine.

[B35] Ojeda H. Deloire A. Carbonneau A. (2001). Influence of water deficits on grape berry growth. *Vitis* 40 141–145.

[B36] Parker A. K. De Cortázar-Atauri I. G. Chuine I. Barbeau G. Bois B. Boursiquot J.-M. (2013). Classification of varieties for their timing of flowering and veraison using a modelling approach: a case study for the grapevine species *Vitis vinifera* L. *Agric. For. Meteorol.* 180 249–264. 10.1016/j.agrformet.2013.06.005

[B37] Parker A. K. de Cortázar-Atauri I. G. Gény L. Spring J. L. Destrac A. Schultz H. (2020). Temperature-based grapevine sugar ripeness modelling for a wide range of *Vitis vinifera* L. cultivars. *Agric. For. Meteorol.* 285:107902 10.1016/j.agrformet.2020.107902

[B38] Parker A. K. De Cortázar-Atauri I. G. van Leeuwen C. Chuine I. (2011). General phenological model to characterise the timing of flowering and veraison of *Vitis vinifera* L. *Aust. J. Grape Wine Res.* 17 206–216. 10.1111/j.1755-0238.2011.00140.x

[B39] Pellegrino A. Lebon E. Simonneau T. Wery J. (2005). Towards a simple indicator of water stress in grapevine (*Vitis vinifera* L.) based on the differential sensitivities of vegetative growth components. *Aust. J. Grape Wine Res.* 11 306–315. 10.1111/j.1755-0238.2005.tb00030.x

[B40] Peyrot des Gachons C. van Leeuwen C. Tominaga T. Soyer J.-P. Gaudillère J.-P. Dubourdieu D. (2005). Influence of water and nitrogen deficit on fruit ripening and aroma potential of *Vitis vinifera* L cv Sauvignon blanc in field conditions. *J. Sci. Food Agric.* 85 73–85. 10.1002/jsfa.1919

[B41] R Core Team (2017). *R: A Language and Environment for Statistical Computing.* Vienna: R Foundation for Statistical Computing.

[B42] Sadras V. O. McCarthy M. G. (2007). Quantifying the dynamics of sugar concentration in berries of *Vitis vinifera* cv. Shiraz: a novel approach based on allometric analysis. *Aust. J. Grape Wine Res.* 13 66–71. 10.1111/j.1755-0238.2007.tb00236.x

[B43] Sadras V. O. Petrie P. R. (2011). Climate shifts in south-eastern Australia: early maturity of Chardonnay, Shiraz and Cabernet Sauvignon is associated with early onset rather than faster ripening. *Aust. J. Grape Wine Res.* 17 199–205. 10.1111/j.1755-0238.2011.00138.x

[B44] Santesteban L. Miranda C. Barbarin I. Royo J. (2015). Application of the measurement of the natural abundance of stable isotopes in viticulture: a review. *Aust. J. Grape Wine Res.* 21 157–167. 10.1111/ajgw.12124

[B45] Santesteban L. G. Royo J. B. (2006). Water status, leaf area and fruit load influence on berry weight and sugar accumulation of cv. ‘Tempranillo’ under semiarid conditions. *Sci. Hortic.* 109 60–65. 10.1016/j.scienta.2006.03.003

[B46] Schultz H. R. (2003). Differences in hydraulic architecture account for near-isohydric and anisohydric behaviour of two field-grown *Vitis vinifera* L. cultivars during drought. *Plant Cell Environ.* 26 1393–1405. 10.1046/j.1365-3040.2003.01064.x

[B47] Shellie K. C. (2014). Water productivity, yield, and berry composition in sustained versus regulated deficit irrigation of merlot grapevines. *Am. J. Enol. Vitic.* 65 197–205. 10.5344/ajev.2014.13112

[B48] Trégoat O. van Leeuwen C. Choné X. Gaudillère J.-P. (2002). The assessment of vine water and nitrogen uptake by means of physiological indicators influence on vine development and berry potential (*Vitis vinifera* L. cv Merlot, 2000, Bordeaux). *J. Int. Sci. Vigne Vin* 36 133–142. 10.20870/oeno-one.2002.36.3.967

[B49] Triboï E. Martre P. Triboï-Blondel A.-M. (2003). Environmentally-induced changes in protein composition in developing grains of wheat are related to changes in total protein content. *J. Exp. Bot.* 54 1731–1742. 10.1093/jxb/erg183 12773520

[B50] van Leeuwen C. Darriet P. (2016). The impact of climate change on viticulture and wine quality. *J. Wine Econ.* 11 150–167.

[B51] van Leeuwen C. Destrac Irvine A. Dubernet M. Duchêne E. Gowdy M. Marguerit E. (2019). An update on the impact of climate change in viticulture and potential adaptations. *Agronomy* 9:514 10.3390/agronomy9090514

[B52] van Leeuwen C. Seguin G. (2006). The concept of terroir in viticulture. *J. Wine Res.* 17 1–10. 10.1080/09571260600633135

[B53] van Leeuwen C. Trégoat O. Choné X. Bois B. Pernet D. Gaudillère J.-P. (2009). Vine water status is a key factor in grape ripening and vintage quality for red Bordeaux wine. How can it be assessed for vineyard management purposes? *J. Int. Sci. Vigne Vin* 43 121–134. 10.20870/oeno-one.2009.43.3.798

[B54] Wolkovich E. M. García de Cortázar-Atauri I. Morales-Castilla I. Nicholas K. A. Lacombe T. (2018). From Pinot to Xinomavro in the world’s future wine-growing regions. *Nat. Clim. Change* 8 29–37. 10.1038/s41558-017-0016-6

[B55] Zufferey V. Murisier F. Schultz H. R. (2000). A model analysis of the photosynthetic response of *Vitis vinifera* L. cvs Riesling and Chasselas leaves in the field: I. Interaction of age, light and temperature. *Vitis* 39 19–26.

