# Supplementary material for: Adapting Wine Grape Ripening to Global Change Requires a Multi-Trait Approach
Source: Front Plant Sci. 2021 Feb 5;12:624867. doi: 10.3389/fpls.2021.624867 (PMC7893094; doi:10.3389/fpls.2021.624867)
Supplement: Supplementary file 1 [file Data_Sheet_1.docx]

**Supplementary Table 1**. Cultivars included in the VitAdapt experimental vineyard.

| Nr. | Cultivar | Colour | Origin | Source | Clone | Year of planting |
| --- | --- | --- | --- | --- | --- | --- |
| 1 | Arinarnoa | red | France | INRA Bordeaux | 723 | 2009 |
| 2 | Assyrtiko | white | Greece | VASSAL | NC | 2009 |
| 3 | BX 9216 (hybrid) | white | INRA Bordeaux | - | NC | 2009 |
| 4 | BX 648 (hybrid) | red | INRA Bordeaux | - | NC | 2009 |
| 5 | Cabernet Franc | red | France | CA 33 | 327 | 2009 |
| 6 | Cabernet-Sauvignon | red | France | CA 33 | 412 | 2009 |
| 7 | Carignan | red | France | ENTAV | 65 | 2009 |
| 8 | Carmenère | red | France | CA 33 | 1059 | 2009 |
| 9 | Castets | red | France | INRA Bordeaux | CC | 2009 |
| 10 | Chardonnay | white | France | INRA Bordeaux | 95 | 2009 |
| 11 | Chenin Blanc | white | France | ENTAV | 1018 | 2009 |
| 12 | Cot | red | France | CA 33 | 1061 | 2009 |
| 13 | Gamay | red | France | ENTAV | 358 | 2009 |
| 14 | Grenache | red | France | ENTAV | 513 | 2009 |
| 15 | Hibernal | white | Geisenheim Germany | - | NC | 2009 |
| 16 | Marselan | red | France | ENTAV | 980 | 2009 |
| 17 | Merlot | red | France | CA 33 | 347 | 2009 |
| 18 | Morrastel | red | France | ENTAV | 949 | 2009 |
| 19 | Mourvèdre | red | France | ENTAV | 369 | 2009 |
| 20 | Muscadelle | white | France | CA 33 | 610 | 2009 |
| 21 | Petit Verdot | red | France | CA 33 | 1058 | 2009 |
| 22 | Petit Arvine | white | Swiss | INRA Bordeaux | NC | 2009 |
| 23 | Pinot Noir | red | France | ENTAV | 667 | 2009 |
| 24 | Prunelard | red | France | Gaillac | CC | 2009 |
| 25 | Riesling | white | France | INRA Bordeaux | 49 | 2009 |
| 26 | Roussanne | white | France | ENTAV | 468 | 2009 |
| 27 | Sangiovese | red | Italy | ENTAV | 903 | 2009 |
| 28 | Saperavi | red | Georgia | INRA Bordeaux | NC | 2009 |
| 29 | Sauvignon Blanc | white | France | CA 33 | 108 | 2009 |
| 30 | Sémillon | white | France | CA 33 | 908 | 2009 |
| 31 | Tempranillo | red | Spain | ENTAV | 771/770 | 2009 |
| 32 | Tinto Cao | red | Portugal | VASSAL | CC | 2009 |
| 33 | Touriga Franca | red | Portugal | VASSAL | NC | 2009 |
| 34 | Touriga Nacional | red | Portugal | INRA Bordeaux | NC | 2009 |
| 35 | Ugni Blanc | white | France | CA 33 | 384 | 2009 |
| 36 | Viognier | white | France | ENTAV | 1051 | 2009 |
|  |  |  |  |  |  |  |
| Not used in this study | |  |  |  |  |  |
| 37 | Alvarinho | white | Spain | - | - | 2010 |
| 38 | Agiorgitiko | red | Greece | - | - | 2009 |
| 39 | Chasselas | white | France | INRA Bordeaux | 887 | 2009 |
| 40 | Colombard | white | France | CA 33 | 605 | 2009 |
| 41 | Cornalin | red | Swiss | INRA Bordeaux | NC | 2009 |
| 42 | Liliorila | white | France | INRA Bordeaux | 734 | 2009 |
| 43 | Mavrud | red | Bulgaria | - | - | 2010 |
| 44 | MPT 3156-26-1 (hybrid) | white | INRA Montpellier | - | NC | 2009 |
| 45 | MPT 3160-12-3 (hybrid) | red | INRA Montpellier | - | NC | 2009 |
| 46 | Nero d'Avola | red | Italy | - | - | NA |
| 47 | Petit Manseng | white | France | CA 64 | 573 | 2009 |
| 48 | Rkatsiteli | white | Georgia | INRA Bordeaux | NC | 2009 |
| 49 | Syrah | red | France | CA 11 | 470 | 2009 |
| 50 | Tannat | red | France | CA 64 | 474 | 2009 |
| 51 | Vinhao | red | Portugal | - | - | 2010 |
| 52 | Xinomavro | red | Greece | - | - | 2010 |

**Supplementary Table 2**. Soil physical and chemical properties of the vineyard soil

|  | 0-70 cm | 70-115 cm | 115-220 cm |
| --- | --- | --- | --- |
| Clay (‰) | 86 | 57 | 112 |
| Fine silt (‰) | 83 | 67 | 72 |
| Coarse silt (‰) | 35 | 28 | 3 |
| Total silt (‰) | 118 | 95 | 75 |
| Fine sand (‰) | 110 | 124 | 89 |
| Coarse sand (‰) | 670 | 710 | 707 |
| Total sand (‰) | 780 | 834 | 796 |
| Organic matter (g kg^-1^) | 15.2 | 12.6 | 13.2 |
| N total | 0.57 | 0.38 | 0.32 |
| P_2_O_5_ (g kg^-1^) | 0.218 | 0.326 | 0.07 |
| K_2_O (g kg^-1^) | 0.085 | 0.073 | 0.082 |
| MgO (g kg^-1^) | 0.031 | 0.033 | 0.032 |
| Cu (mg kg^-1^) | 9.05 | 1.54 | 0.49 |
| Mn (mg kg^-1^) | 7.06 | 0.19 | 0.06 |
| pH-H_2_O | 6.06 | 6.37 | 5.79 |
| pH-KCl | 4.8 | 5.14 | 4.41 |
| CaO (g kg^-1^) | 0.52 | 0.56 | 0.38 |
| CEC-Metson (Cmol+ kg^-1^) | 3.6 | 3.2 | 3.1 |
| Ca/CEC | 52 | 63 | 44 |
| S/CEC | 61 | 73 | 55 |
| C/N | 15.63 | 19.31 | 21.84 |

**Supplementary Table 3**. Sugar concentration (g L^-1^) and content (mg berry^-1^) at mid-véraison for all cultivars. The six clusters identified are as in Fig 3. DOY = day of the year. CV pooled = pooled coefficient of variation. SD pooled = pooled standard deviation. 95% CI = average 95% confidence interval with standard deviation.


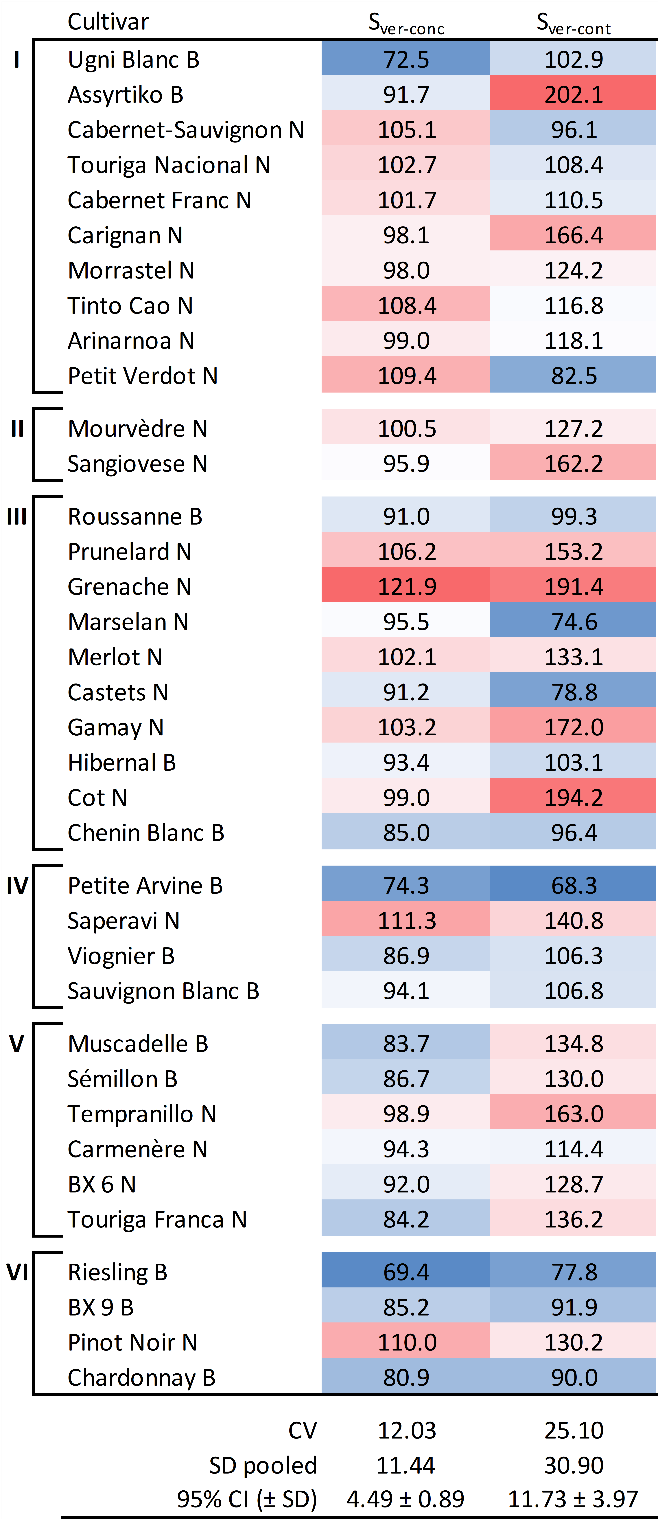


**Supplementary Table 4**. Comparison of goodness-of-fit and predictive power of the sigmoidal model for the 36 grape cultivars. Means are followed by standard deviations.

| Cultivar | r^2^ (concentration) | r^2^ (content) | RMSE (g L^-1^) | RMSE (mg berry^-1^) |  |
| --- | --- | --- | --- | --- | --- |
| Arinarnoa N* | 0.98 ± 0.01 | 0.94 ± 0.06 | 4.22 ± 1.06 | 16.32 ± 8.74 |  |
| Assyrtiko B | 0.98 ± 0.02 | 0.96 ± 0.05 | 4.45 ± 2.01 | 29.49 ± 16.32 |  |
| BX 6 N | 0.98 ± 0.02 | 0.97 ± 0.03 | 4.85 ± 1.83 | 13.23 ± 7.48 |  |
| BX 9 B | 0.99 ± 0.01 | 0.96 ± 0.04 | 3.58 ± 1.52 | 16.82 ± 7.19 |  |
| Cabernet Franc N | 0.98 ± 0.03 | 0.96 ± 0.03 | 3.80 ± 1.77 | 20.43 ± 8.77 |  |
| Cabernet-Sauvignon N | 0.99 ± 0.01 | 0.95 ± 0.04 | 3.62 ± 0.97 | 19.12 ± 8.28 |  |
| Carignan N | 0.98 ± 0.02 | 0.96 ± 0.04 | 4.16 ± 1.39 | 11.63 ± 6.57 |  |
| Carmenère N | 0.98 ± 0.01 | 0.95 ± 0.06 | 4.10 ± 1.56 | 11.61 ± 6.31 |  |
| Castets N | 0.99 ± 0.01 | 0.97 ± 0.02 | 3.75 ± 1.36 | 11.89 ± 4.70 |  |
| Chardonnay B | 0.99 ± 0.01 | 0.98 ± 0.02 | 4.01 ± 1.29 | 12.18 ± 4.66 |  |
| Chenin Blanc B | 0.99 ± 0.01 | 0.96 ± 0.05 | 3.51 ± 1.56 | 22.30 ± 10.23 |  |
| Cot N | 0.99 ± 0.01 | 0.94 ± 0.06 | 4.18 ± 1.20 | 12.74 ± 6.05 |  |
| Gamay N | 0.98 ± 0.03 | 0.92 ± 0.11 | 4.10 ± 2.03 | 17.33 ± 10.25 |  |
| Grenache N | 0.96 ± 0.05 | 0.95 ± 0.05 | 4.44 ± 2.26 | 18.87 ± 9.16 |  |
| Hibernal B | 0.99 ± 0.02 | 0.97 ± 0.03 | 3.51 ± 1.93 | 10.99 ± 6.16 |  |
| Marselan N | 0.99 ± 0.01 | 0.97 ± 0.03 | 4.21 ± 1.62 | 9.73 ± 3.71 |  |
| Merlot N | 0.99 ± 0.01 | 0.96 ± 0.04 | 3.30 ± 0.93 | 14.82 ± 7.50 |  |
| Morrastel N | 0.98 ± 0.03 | 0.94 ± 0.08 | 3.67 ± 1.32 | 14.19 ± 8.73 |  |
| Mourvèdre N | 0.96 ± 0.05 | 0.94 ± 0.05 | 4.61 ± 1.94 | 20.21 ± 10.16 |  |
| Muscadelle B | 0.98 ± 0.02 | 0.95 ± 0.05 | 5.03 ± 1.87 | 14.85 ± 7.12 |  |
| Petit Verdot N | 0.99 ± 0.02 | 0.97 ± 0.05 | 4.58 ± 2.88 | 9.89 ± 6.03 |  |
| Petite Arvine B | 0.99 ± 0.01 | 0.95 ± 0.06 | 3.67 ± 1.50 | 12.08 ± 7.48 |  |
| Pinot Noir N | 0.98 ± 0.02 | 0.93 ± 0.07 | 3.92 ± 1.57 | 18.50 ± 10.90 |  |
| Prunelard N | 0.97 ± 0.05 | 0.92 ± 0.10 | 5.00 ± 1.90 | 11.80 ± 5.68 |  |
| Riesling B | 0.99 ± 0.03 | 0.97 ± 0.04 | 3.81 ± 2.49 | 12.29 ± 8.11 |  |
| Roussanne B | 0.98 ± 0.01 | 0.96 ± 0.04 | 4.97 ± 1.36 | 15.17 ± 7.92 |  |
| Sangiovese N | 0.99 ± 0.01 | 0.93 ± 0.06 | 3.71 ± 1.24 | 24.13 ± 10.95 |  |
| Saperavi N | 0.98 ± 0.02 | 0.97 ± 0.04 | 5.56 ± 3.13 | 11.03 ± 5.34 |  |
| Sauvignon Blanc B | 0.99 ± 0.01 | 0.98 ± 0.02 | 4.78 ± 1.81 | 14.19 ± 4.72 |  |
| Sémillon B | 0.98 ± 0.02 | 0.96 ± 0.05 | 4.78 ± 1.80 | 21.63 ± 12.51 |  |
| Tempranillo N | 0.98 ± 0.01 | 0.94 ± 0.07 | 3.97 ± 1.09 | 14.94 ± 9.00 |  |
| Tinto Cao N | 0.98 ± 0.01 | 0.96 ± 0.04 | 3.75 ± 1.37 | 18.56 ± 9.76 |  |
| Touriga Franca N | 0.98 ± 0.02 | 0.96 ± 0.03 | 3.58 ± 1.91 | 16.45 ± 6.75 |  |
| Touriga Nacional N | 0.98 ± 0.01 | 0.92 ± 0.08 | 3.72 ± 1.45 | 16.16 ± 6.78 |  |
| Ugni Blanc B | 0.99 ± 0.01 | 0.96 ± 0.04 | 4.41 ± 1.37 | 19.92 ± 8.21 |  |
| Viognier B | 0.99 ± 0.01 | 0.97 ± 0.03 | 4.86 ± 1.73 | 11.93 ± 8.84 |  |
| *N = red grape cultivars, B = white grape cultivars | | | | | |

**Supplementary Figure 1.** Sugar accumulation dynamics of the 36 grape cultivars included in this study from 2012-2018. The curves represent a single year and were drawn from traits averaged over the four blocks.
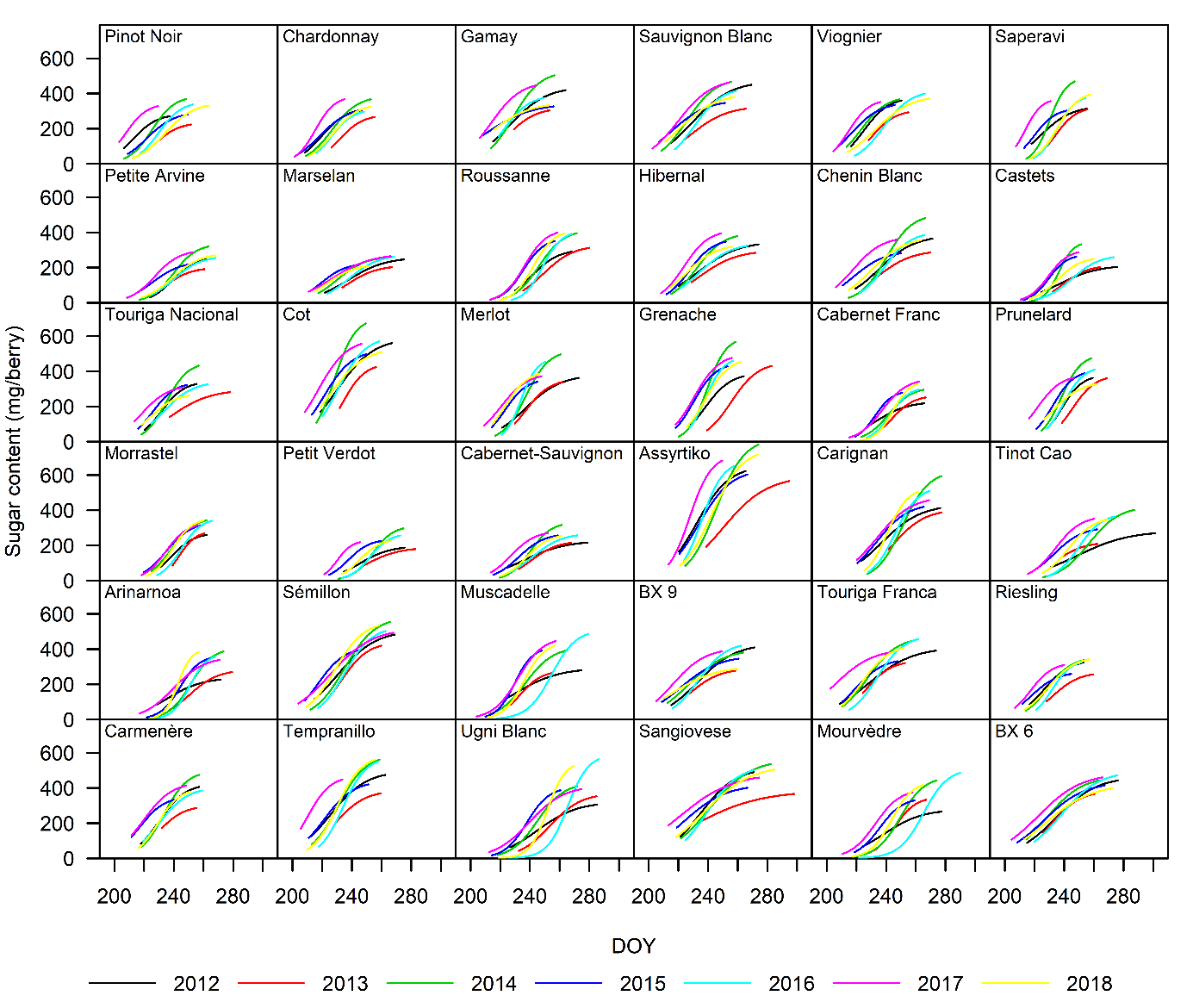


**Supplementary Table 5.** Means and standard deviations of the key sugar accumulation traits t_ver_ (DOY), Dur_cont_ (days) and S_95-conc_ (g L^-1^) for the 36 cultivars. Blue and red cells are increasingly lower and higher values, respectively. Letters represent Tukey’s HSD pairwise comparisons across cultivar means.

| Cultivar | t_ver_ | | |  | Dur_cont_ | | |  | S_95-conc_ | | |  |
| --- | --- | --- | --- | --- | --- | --- | --- | --- | --- | --- | --- | --- |
| Chardonnay | 211.2 | ± | 7.8 | a | 37.1 | ± | 7.5 | abcdef | 214.7 | ± | 14.1 | cdefghi |
| Pinot Noir | 211.5 | ± | 7.7 | a | 35.5 | ± | 15.4 | abcd | 221.7 | ± | 18.5 | ghijk |
| Touriga Franca | 212.1 | ± | 6.8 | ab | 44.0 | ± | 15.2 | bcdefghi | 177.6 | ± | 20.3 | a |
| Sauvignon Blanc | 212.3 | ± | 7.1 | ab | 46.7 | ± | 9.3 | defghij | 237.3 | ± | 18.7 | jk |
| Sémillon | 212.9 | ± | 6.8 | abc | 48.2 | ± | 14.6 | fghij | 195.1 | ± | 17.9 | abcd |
| BX 9 | 213.7 | ± | 6.9 | abcd | 47.6 | ± | 11.2 | efghij | 204.2 | ± | 11.5 | bcdefgh |
| Tempranillo | 214.4 | ± | 7.6 | abcde | 40.0 | ± | 11.3 | abcdefg | 200.5 | ± | 26.9 | bcdefg |
| BX 6 | 214.7 | ± | 7.0 | abcdef | 54.1 | ± | 16.2 | hij | 214.8 | ± | 17.1 | cdefghij |
| Viognier | 214.8 | ± | 7.7 | abcdef | 38.3 | ± | 12.0 | abcdef | 237.2 | ± | 19.5 | ijk |
| Muscadelle | 215.0 | ± | 7.5 | abcdef | 47.4 | ± | 16.4 | efghij | 198.1 | ± | 20.3 | abcdef |
| Gamay | 215.3 | ± | 7.3 | abcdef | 38.5 | ± | 13.2 | abcdef | 217.5 | ± | 20.0 | defghij |
| Riesling | 216.1 | ± | 6.7 | abcdef | 35.3 | ± | 5.8 | abcd | 187.0 | ± | 12.4 | ab |
| Chenin Blanc | 217.0 | ± | 7.9 | abcdefg | 45.3 | ± | 8.4 | cdefghij | 213.3 | ± | 15.4 | cdefgh |
| Saperavi | 217.0 | ± | 6.2 | abcdefg | 32.7 | ± | 10.1 | ab | 241.6 | ± | 23.2 | k |
| Hibernal | 217.3 | ± | 6.7 | abcdefg | 43.9 | ± | 7.9 | bcdefghi | 208.5 | ± | 18.6 | bcdefgh |
| Cot | 218.1 | ± | 7.0 | abcdefg | 37.0 | ± | 10.5 | abcdef | 211.9 | ± | 17.1 | cdefgh |
| Carmenère | 218.2 | ± | 7.1 | abcdefg | 34.5 | ± | 7.3 | abc | 197.5 | ± | 15.5 | abcde |
| Merlot | 218.5 | ± | 6.5 | abcdefgh | 35.7 | ± | 10.1 | abcd | 225.5 | ± | 18.2 | hijk |
| Castets | 219.3 | ± | 7.5 | bcdefghi | 41.4 | ± | 11.3 | abcdefg | 208.6 | ± | 27.1 | bcdefgh |
| Marselan | 219.3 | ± | 7.6 | bcdefghi | 42.8 | ± | 13.3 | abcdefgh | 220.2 | ± | 19.7 | fghijk |
| Petite Arvine | 220.3 | ± | 7.4 | cdefghij | 41.4 | ± | 9.8 | abcdefg | 223.7 | ± | 20.2 | hijk |
| Mourvèdre | 220.4 | ± | 6.8 | defghij | 50.7 | ± | 16.9 | ghij | 201.1 | ± | 32.3 | bcdefg |
| Ugni Blanc | 221.3 | ± | 6.9 | efghij | 55.5 | ± | 12.4 | ij | 208.0 | ± | 32.0 | bcdefgh |
| Touriga Nacional | 221.3 | ± | 7.7 | efghij | 36.0 | ± | 6.8 | abcde | 212.7 | ± | 17.6 | cdefgh |
| Cabernet-Sauvignon | 221.7 | ± | 6.2 | efghij | 44.3 | ± | 10.2 | bcdefghi | 207.3 | ± | 19.9 | bcdefgh |
| Sangiovese | 221.9 | ± | 6.6 | fghij | 56.4 | ± | 12.8 | j | 206.3 | ± | 25.0 | bcdefgh |
| Assyrtiko | 223.6 | ± | 7.8 | ghij | 45.3 | ± | 11.5 | cdefghij | 207.0 | ± | 28.6 | bcdefgh |
| Roussanne | 223.8 | ± | 7.4 | ghij | 43.0 | ± | 8.1 | abcdefgh | 218.9 | ± | 23.6 | efghij |
| Cabernet Franc | 224.4 | ± | 6.5 | ghij | 38.0 | ± | 9.4 | abcdef | 210.4 | ± | 21.3 | cdefgh |
| Arinarnoa | 225.7 | ± | 6.4 | hijk | 44.1 | ± | 10.1 | bcdefghi | 210.2 | ± | 27.9 | cdefgh |
| Grenache | 225.7 | ± | 7.4 | hijk | 35.7 | ± | 7.2 | abcd | 212.4 | ± | 20.7 | cdefgh |
| Morrastel | 226.5 | ± | 7.5 | ijk | 35.1 | ± | 9.1 | abcd | 193.2 | ± | 26.0 | abc |
| Carignan | 226.7 | ± | 7.6 | ijk | 44.3 | ± | 9.4 | bcdefghi | 199.3 | ± | 23.0 | abcdefg |
| Tinto Cao | 226.7 | ± | 7.6 | ijk | 47.6 | ± | 17.1 | efghij | 215.9 | ± | 28.1 | defghij |
| Prunelard | 226.9 | ± | 6.9 | jk | 31.8 | ± | 6.4 | a | 208.3 | ± | 17.0 | bcdefgh |
| Petit Verdot | 232.4 | ± | 8.0 | k | 35.5 | ± | 8.6 | abcd | 215.9 | ± | 30.6 | defghij |

**Supplementary Table 6.** Regression coefficients for each cultivar of the significant variables in the models for the dependent variable Dur_cont_.

| Cultivar | adj. r^2^ | RMSE | RR_f-v_ | T_f-v_ | PAR_f-v_ | T_v-95_ | PAR_v-95_ | δ^13^C | BW_v_ | t_ver_ |
| --- | --- | --- | --- | --- | --- | --- | --- | --- | --- | --- |
| Arinarnoa | 0.68 | 5.7 |  | -5.99 |  | -6.96 |  |  |  | -0.69 |
| Assyrtiko | 0.80 | 5.2 | -0.10 |  |  |  | -0.07 |  | 10.09 |  |
| BX 6 | 0.86 | 6.0 | -0.09 |  |  |  | -0.16 |  | 21.21 | -0.70 |
| BX 9 | 0.84 | 4.5 | -0.06 | -6.10 |  |  | -0.12 |  |  | -0.81 |
| Carignan | 0.67 | 5.4 |  | -5.60 |  | -3.91 |  |  |  | -0.81 |
| Carmenère | 0.77 | 3.5 | -0.11 |  | -0.05 |  |  |  | 14.93 |  |
| Castets | 0.42 | 8.6 | -0.10 | -5.58 |  |  | -0.06 |  |  |  |
| Cabernet Franc | 0.63 | 5.7 |  |  |  |  | -0.08 | 1.95 | 19.23 | -1.06 |
| Chardonnay | 0.73 | 3.9 | -0.11 |  |  |  | -0.06 |  | 19.55 |  |
| Chenin Blanc | 0.58 | 5.4 | -0.11 |  |  |  | -0.07 |  | 22.73 |  |
| Cot | 0.70 | 5.7 | -0.08 | -4.86 |  |  | -0.10 |  |  | -0.71 |
| Cabernet-Sauvignon | 0.74 | 5.1 | -0.14 |  |  |  | -0.08 |  | 27.00 |  |
| Gamay | 0.66 | 7.7 | -0.13 |  | 0.04 |  | -0.10 |  | 13.16 |  |
| Grenache | 0.44 | 5.4 |  |  |  |  | -0.07 |  |  | -0.80 |
| Hibernal | 0.51 | 5.5 | -0.10 |  |  |  | -0.09 |  | 23.02 |  |
| Marselan | 0.81 | 5.8 |  | -9.89 |  |  | -0.14 |  |  | -1.70 |
| Merlot | 0.84 | 4.0 |  | -10.03 |  |  | -0.10 |  |  | -1.20 |
| Morrastel | 0.76 | 4.4 | -0.11 |  |  |  | -0.07 |  | 19.97 | -1.09 |
| Mourvèdre | 0.89 | 5.7 | -0.14 | -7.64 |  |  | -0.16 |  | 11.87 | -1.65 |
| Muscadelle | 0.82 | 6.9 | -0.13 |  |  |  | -0.15 |  | 28.30 |  |
| Petite Arvine | 0.82 | 4.2 | -0.12 |  |  |  | -0.10 |  | 25.72 | -0.61 |
| Pinot Noir | 0.59 | 9.8 | -0.13 |  |  |  | -0.11 |  | 33.32 | 0.86 |
| Prunelard | 0.30 | 5.4 |  |  |  |  | -0.03 | -2.05 |  |  |
| Petit Verdot | 0.42 | 6.6 | -0.11 |  |  | -2.57 |  |  | 35.09 |  |
| Riesling | 0.70 | 3.1 | -0.09 |  |  |  | -0.07 |  | 22.60 |  |
| Roussanne | 0.34 | 6.6 |  |  |  | -2.92 |  |  | 12.29 |  |
| Sangiovese | 0.81 | 5.5 |  |  |  |  | -0.13 | 1.72 | 7.52 | -1.22 |
| Saperavi | 0.69 | 5.7 | -0.10 | -4.66 |  |  | -0.11 |  |  |  |
| Sauvignon Blanc | 0.63 | 5.7 | -0.04 | -4.40 |  |  | -0.13 |  |  | -0.65 |
| Sémillon | 0.78 | 6.8 |  | -6.76 |  |  | -0.15 |  |  | -1.13 |
| Tinto Cao | 0.90 | 5.4 | -0.06 | -10.94 |  |  | -0.16 |  |  | -1.90 |
| Tempranillo | 0.74 | 5.8 | -0.24 |  | -0.04 |  | -0.09 |  | 16.48 |  |
| Touriga Franca | 0.84 | 6.1 | -0.04 | -8.20 |  |  | -0.12 |  |  | -0.54 |
| Touriga Nacional | 0.45 | 5.0 |  |  |  |  | -0.03 | 1.06 | 9.44 |  |
| Ugni Blanc | 0.74 | 6.3 | -0.12 |  |  | -8.22 |  |  | 24.52 | -0.48 |
| Viognier | 0.73 | 6.2 | -0.16 | -4.84 |  |  | -0.12 | -4.13 |  |  |

**Supplementary Table 7.** Regression coefficients for each cultivar of the significant variables in the models for the dependent variable S_95-cont_.

| Cultivar | adj. r^2^ | RMSE | Dur_cont_ | RR_f-v_ | T_f-v_ | PAR_f-v_ | T_v-95_ | PAR_v-95_ | δ^13^C | (δ^13^C)^2^ | BW_v_ | t_ver_ |
| --- | --- | --- | --- | --- | --- | --- | --- | --- | --- | --- | --- | --- |
| Arinarnoa | 0.69 | 35.7 | 1.30 |  |  | -0.39 |  | 0.40 | -777.92 | -15.29 |  |  |
| Assyrtiko | 0.36 | 71.8 | 2.70 |  |  |  |  |  |  |  | 78.17 | -5.32 |
| BX 6 | 0.74 | 34.9 |  |  |  | 0.42 |  | -0.43 |  |  | 243.66 |  |
| BX 9 | 0.77 | 31.7 |  | -0.47 |  |  |  | 0.21 |  |  | 229.32 |  |
| Carignan | 0.75 | 43.8 |  | -1.63 |  | -0.81 |  | 0.54 | -34.18 |  | 204.81 |  |
| Carmenère | 0.60 | 44.0 |  |  |  | -0.26 |  |  |  |  | 125.40 | -5.19 |
| Castets | 0.71 | 29.4 |  | -0.64 |  |  |  |  | -26.16 |  | 209.24 |  |
| Cabernet Franc | 0.64 | 36.3 |  | -0.77 |  | -0.41 |  |  | -34.12 |  | 111.13 |  |
| Chardonnay | 0.81 | 19.5 | 1.94 |  | -29.53 |  |  |  | -27.11 |  |  | -2.99 |
| Chenin Blanc | 0.75 | 38.0 | 2.48 | -0.34 |  |  |  |  | -337.67 | -6.57 | 246.68 |  |
| Cot | 0.69 | 49.7 |  | -1.07 |  |  | -25.81 |  | -20.35 |  | 188.11 |  |
| Cabernet-Sauvignon | 0.62 | 25.8 |  |  |  |  |  |  | -392.50 | -7.77 | 119.79 | -2.64 |
| Gamay | 0.78 | 45.6 | 2.91 |  |  |  |  |  |  |  | 158.24 |  |
| Grenache | 0.60 | 44.1 |  |  |  |  | 15.42 |  | -15.84 |  | 204.86 |  |
| Hibernal | 0.79 | 27.8 |  | -0.72 |  |  |  |  | -27.85 |  | 284.23 |  |
| Marselan | 0.84 | 16.8 |  | -0.35 | 14.31 |  |  |  |  |  | 253.37 |  |
| Merlot | 0.81 | 27.9 |  | -0.75 |  |  |  |  | -9.04 |  | 242.33 |  |
| Morrastel | 0.62 | 29.6 |  | -0.34 | 21.98 |  | 27.94 |  | -14.79 |  | 132.81 |  |
| Mourvèdre | 0.71 | 52.0 | 3.68 |  |  |  |  |  | -641.41 | -12.56 | 97.64 |  |
| Muscadelle | 0.78 | 49.1 | 4.50 | -0.86 |  |  |  |  | -45.07 |  |  |  |
| Petite Arvine | 0.63 | 28.9 | 2.49 |  |  | -0.28 |  | 0.38 |  |  | 59.12 |  |
| Pinot Noir | 0.76 | 28.2 | 1.82 |  |  |  |  |  |  |  | 184.29 |  |
| Prunelard | 0.49 | 39.2 |  |  | -64.60 |  | -11.93 |  | -381.23 | -7.69 |  |  |
| Petit Verdot | 0.66 | 26.1 | 4.11 |  |  | -0.32 |  | 0.27 | -384.12 | -7.62 |  |  |
| Riesling | 0.71 | 21.3 |  |  |  | -0.28 | 9.23 |  | -573.80 | -11.58 | 100.79 | -3.76 |
| Roussanne | 0.79 | 26.2 | 4.28 |  |  |  | 40.00 |  | -13.78 |  | 98.74 |  |
| Sangiovese | 0.56 | 59.8 |  | -0.96 |  |  |  |  |  |  | 227.72 |  |
| Saperavi | 0.71 | 34.7 |  | -0.79 |  | -0.33 |  |  | -256.69 | -4.82 | 200.23 |  |
| Sauvignon Blanc | 0.80 | 29.4 | 2.86 |  |  |  |  | 0.65 | -12.64 |  | 203.88 |  |
| Sémillon | 0.39 | 71.1 | 2.53 |  |  |  | 29.83 |  | -22.01 |  | 92.17 |  |
| Tinto Cao | 0.78 | 31.9 | 3.24 |  |  | -0.38 |  | 0.75 |  |  | 94.64 |  |
| Tempranillo | 0.77 | 45.4 | 6.82 |  |  | -0.72 |  | 0.82 | -442.22 | -8.30 |  |  |
| Touriga Franca | 0.60 | 49.7 | 3.65 |  |  |  |  |  | -750.01 | -15.27 | 86.68 |  |
| Touriga Nacional | 0.59 | 40.4 |  |  |  |  |  |  |  |  | 175.94 | -3.86 |
| Ugni Blanc | 0.63 | 60.8 | 3.53 | -2.02 |  | -0.99 |  |  | -2383.98 | -47.39 |  |  |
| Viognier | 0.89 | 13.2 | 2.65 |  | -32.96 |  |  |  | -9.17 |  |  | -1.74 |

**Supplementary Table 8.** Regression coefficients for each cultivar of the significant variables in the models for the dependent variable S_95-conc_.

| Cultivar | adj. r^2^ | RMSE | RR_f-v_ | T_f-v_ | PAR_f-v_ | T_v-95_ | PAR_v-95_ | δ^13^C | (δ^13^C)^2^ | BW_v_ | t_ver_ |
| --- | --- | --- | --- | --- | --- | --- | --- | --- | --- | --- | --- |
| Arinarnoa | 0.87 | 10.2 | -0.40 | 15.45 |  |  | 0.19 | -185.89 | -3.64 |  |  |
| Assyrtiko | 0.59 | 18.3 | -0.35 | 19.88 |  |  | 0.13 |  |  |  |  |
| BX 6 | 0.43 | 12.9 | -0.12 | 10.11 |  |  | 0.07 |  |  |  |  |
| BX 9 | 0.79 | 5.3 | -0.19 |  | -0.06 |  |  | 56.98 | 1.17 |  |  |
| Carignan | 0.77 | 11.0 | -0.14 | 19.67 |  |  | 0.16 |  |  |  |  |
| Carmenère | 0.54 | 10.5 | -0.19 |  | -0.08 |  |  | -8.16 |  |  |  |
| Castets | 0.76 | 13.4 | -0.52 |  | -0.16 |  |  | -17.02 |  |  |  |
| Cabernet Franc | 0.64 | 12.8 | -0.32 |  | -0.16 |  |  | -110.36 | -2.02 |  |  |
| Chardonnay | 0.72 | 7.5 | -0.27 |  | -0.09 |  |  | -6.79 |  | 17.12 |  |
| Chenin Blanc | 0.69 | 8.6 | -0.25 |  | -0.07 |  |  | -102.32 | -2.01 | 14.03 |  |
| Cot | 0.88 | 6.0 | -0.31 |  | -0.13 |  |  | -7.22 |  |  | -0.43 |
| Cabernet-Sauvignon | 0.75 | 9.9 | -0.34 |  | -0.15 |  |  | -182.45 | -3.44 |  |  |
| Gamay | 0.51 | 14.0 | -0.14 | 12.43 |  |  | 0.14 |  |  | 23.57 |  |
| Grenache | 0.69 | 11.6 | -0.12 |  |  | 5.28 |  | -91.52 | -1.79 |  | -1.26 |
| Hibernal | 0.73 | 9.7 | -0.32 |  |  | -6.02 |  | -76.15 | -1.39 | 34.25 |  |
| Marselan | 0.73 | 10.3 | -0.36 |  | -0.14 |  |  | -93.51 | -1.76 |  |  |
| Merlot | 0.87 | 6.6 | -0.38 |  | -0.10 |  |  | -4.66 |  |  |  |
| Morrastel | 0.74 | 13.3 | -0.27 | 16.57 |  |  | 0.18 | -186.70 | -3.71 |  |  |
| Mourvèdre | 0.76 | 15.7 | -0.55 | 21.33 |  |  |  | -135.87 | -2.73 |  | -1.75 |
| Muscadelle | 0.80 | 9.2 | -0.49 |  | -0.12 |  |  | -12.17 |  | 22.53 |  |
| Petite Arvine | 0.81 | 8.8 | -0.41 |  | -0.22 |  |  | -5.11 |  |  |  |
| Pinot Noir | 0.47 | 13.5 | -0.23 |  | -0.09 |  | 0.07 | -10.07 |  | 29.92 |  |
| Prunelard | 0.52 | 11.8 | -0.20 |  |  |  |  | -179.67 | -3.76 |  |  |
| Petit Verdot | 0.60 | 19.3 |  | 17.41 |  | 6.94 |  | -425.64 | -8.69 |  |  |
| Riesling | 0.77 | 5.9 | -0.17 |  | -0.17 |  |  |  |  |  |  |
| Roussanne | 0.70 | 13.0 | -0.53 |  | -0.14 |  |  | -96.42 | -1.82 |  |  |
| Sangiovese | 0.66 | 14.5 | -0.40 | 10.72 |  |  |  |  |  |  |  |
| Saperavi | 0.66 | 13.5 | -0.36 |  | -0.21 |  | 0.13 |  |  | 31.83 |  |
| Sauvignon Blanc | 0.77 | 9.1 | -0.15 |  |  | 11.28 |  | -10.64 |  | 32.54 |  |
| Sémillon | 0.59 | 11.5 | -0.31 |  | -0.14 |  |  | -94.92 | -1.83 |  |  |
| Tinto Cao | 0.86 | 10.7 | -0.22 |  |  |  |  | -227.53 | -4.23 |  | -1.39 |
| Tempranillo | 0.75 | 13.5 | -0.51 |  | -0.22 |  |  | -148.02 | -2.77 |  |  |
| Touriga Franca | 0.55 | 13.7 |  |  |  | 7.38 |  | -196.44 | -4.09 |  | -0.93 |
| Touriga Nacional | 0.49 | 12.5 | -0.32 |  | -0.14 |  |  | -4.26 |  | 25.09 |  |
| Ugni Blanc | 0.76 | 15.6 | -0.88 |  | -0.41 |  |  | -331.74 | -6.36 |  | -0.94 |
| Viognier | 0.87 | 6.9 | -0.20 |  | -0.15 |  |  |  |  |  | -1.71 |
